# Two models of inescapable stress increase *tph2* mRNA expression in the anxiety-related dorsomedial part of the dorsal raphe nucleus

**DOI:** 10.1016/j.ynstr.2018.01.003

**Published:** 2018-01-17

**Authors:** Nina C. Donner, Kenneth H. Kubala, James E. Hassell Jr., Margaret W. Lieb, Kadi T. Nguyen, Jared D. Heinze, Robert C. Drugan, Steven F. Maier, Christopher A. Lowry

**Affiliations:** a Department of Integrative Physiology and Center for Neuroscience, University of Colorado Boulder, Boulder, CO 80309, USA; b Department of Psychology and Neuroscience and Center for Neuroscience, University of Colorado Boulder, Boulder, CO 80302, USA; c Department of Psychology, University of New Hampshire, Durham, NH 03824, USA; d Department of Physical Medicine & Rehabilitation and Center for Neuroscience, University of Colorado Anschutz Medical Campus, Aurora, CO 80045, USA; e Rocky Mountain Mental Illness Research Education and Clinical Center, Denver Veterans Affairs Medical Center, Denver, CO 80220, USA; f Military and Veteran Microbiome: Consortium for Research and Education, Denver, CO 80220, USA

**Keywords:** Anxiety, Dorsal raphe nucleus, Inescapable stress, Inflammation, Tryptophan hydroxylase

## Abstract

Expression of TPH2, the rate-limiting enzyme for brain serotonin synthesis, is elevated in the dorsal raphe nucleus (DR) of depressed suicide victims. One hypothesis is that this increase in TPH2 expression is stress-induced. Here, we used an established animal model to address whether exposure to an acute stressor, inescapable tail shock (IS), increases *tph2* mRNA and Tph2 protein expression, and if IS sensitizes the DR to a subsequent, heterotypic stressor. In *Experiment 1*, we measured *tph2* mRNA expression 4 h after IS or home cage (HC) control conditions in male rats, using *in situ* hybridization histochemistry. In *Experiment 2*, we measured Tph2 protein expression 12 h or 24 h after IS using western blot. In *Experiment 3*, we measured *tph2* mRNA expression following IS on Day 1, and cold swim stress (10 min, 15 °C) on Day 2. Inescapable tail shock was sufficient to increase *tph2* mRNA expression 4 h and 28 h later, selectively in the dorsomedial DR (caudal aspect of the dorsal DR, cDRD; an area just rostral to the caudal DR, DRC) and increased Tph2 protein expression in the DRD (rostral and caudal aspects of the dorsal DR combined) 24 h later. Cold swim increased *tph2* mRNA expression in the dorsomedial DR (cDRD) 4 h later. These effects were associated with increased immobility during cold swim, elevated plasma corticosterone, and a proinflammatory plasma cytokine milieu (increased interleukin (IL)-6, decreased IL-10). Our data demonstrate that two models of inescapable stress, IS and cold swim, increase *tph2* mRNA expression selectively in the anxiety-related dorsomedial DR (cDRD).

## List of abbreviations

5-HT: 5-hydroxytryptamine, serotonin
5-HTP: 5-hydroxytryptophan
ANOVA: analysis of variance
B9: supralemniscal serotonergic cell group
BLA: basolateral amygdala
BNST: bed nucleus of the stria terminalis
CeA: central amygdaloid nucleus
cDRD: caudal aspect of the dorsal raphe nucleus, dorsal part (dorsomedial dorsal raphe nucleus)
cDRV: caudal aspect of the dorsal raphe nucleus, ventral part
cMnR: caudal aspect of the median raphe nucleus
CRF: corticotropin-releasing factor
CRFR2: corticotropin-releasing factor receptor type 2
CV: coefficient of variation
DR: dorsal raphe nucleus
DRC: dorsal raphe nucleus, caudal part
DRD: dorsal raphe nucleus, dorsal part
DRI: dorsal raphe nucleus, interfascicular part
DRV: dorsal raphe nucleus, ventral part
DRVL: dorsal raphe nucleus, ventrolateral part
EDTA: ethylenediaminetetraacetic acid
HC: home cage control group
IL: interleukin
IS: inescapable tail shock
ISHH: *in situ* hybridization histochemistry
LMM: linear mixed model
LSD: least significant difference
mCPP: m-chlorophenylpiperazine
MnR: median raphe nucleus
PBS: phosphate-buffered saline
PMRF: pontomesencephalic reticular formation
rDRD: rostral aspect of the dorsal raphe nucleus, dorsal part
rDRV: rostral aspect of the dorsal raphe nucleus, ventral part
rMnR: rostral aspect of the median raphe nucleus
S: cold swim stress
SEM: standard error of the mean
SSRI: selective serotonin reuptake inhibitor
*tph2*: tryptophan hydroxylase 2 gene, nonhuman
*TPH2*: tryptophan hydroxylase 2 gene, human
Tph2: tryptophan hydroxylase 2 protein, nonhuman
TPH2: tryptophan hydroxylase 2 protein, human
VLPAG: ventrolateral periaqueductal gray

## 1 Introduction

Anxiety disorders, stress- or trauma-related disorders, and affective disorders are common, with a life-time prevalence of up to 20% of the population, and represent a significant social and economic burden (Kessler et al., 2005, Wittchen et al., 2011). A major risk factor for the development of anxiety and affective disorders is exposure to stressful life events (Zavos et al., 2012, Pemberton and Fuller Tyszkiewicz, 2016). One mechanism through which adverse events may affect risk for stress-related psychiatric disorders is through effects on brainstem serotonergic systems (Valentino and Commons, 2005).

One well-studied model of how uncontrollable stress can lead to dysregulation of neuronal circuits and behavioral responses is inescapable tail shock (IS) in adult male rats (Maier and Watkins, 2005). Behavioral consequences of IS have been shown to depend on sensitization of serotonergic neurons in the dorsal raphe nucleus (DR). Inescapable tail shock leads to large increases in extracellular 5-hydroxytryptamine (5-HT, serotonin) in the DR (Maswood et al., 1998), and increases in anxiety-related behaviors measured 24 h later, such as exaggerated fear conditioning and escape deficits in a shuttle box escape task, behavioral consequences that are absent if the DR is lesioned or inhibited during IS (Maier et al., 1993, Maier et al., 1995). Hyperexcitability of DR serotonergic neurons, measured 24 h following IS, is thought to be dependent on a functional desensitization of inhibitory 5-HT_1A_ autoreceptors in the dorsal DR (DRD) (Rozeske et al., 2011), leading to exaggerated release of serotonin in forebrain circuits mediating anxiety and fear responses (Amat et al., 1998, Christianson et al., 2010).

The effects of IS on serotonergic gene and protein expression in the DR are not yet known. Expression of *tph2,* the gene encoding tryptophan hydroxylase 2 (Tph2), the rate-limiting enzyme in brain serotonin synthesis (Walther et al., 2003), is of particular interest because in rodents it is sensitive to developmental stressors (Gardner et al., 2009a, Gardner et al., 2009b; Lukkes et al., 2013) and glucocorticoid stress hormones (Donner et al., 2012b). In humans, elevated *TPH2* mRNA expression (Bach-Mizrachi et al., 2006, Bach-Mizrachi et al., 2008) and increased TPH2 immunoreactivity has been reported within subregions in the DR of depressed suicides (Boldrini et al., 2005, Bonkale et al., 2006), specifically the dorsomedial DR (Bonkale et al., 2006).

Therefore, we tested the hypothesis that IS leads to increased *tph2* mRNA and Tph2 protein expression within the rat dorsomedial DR (cDRD), and that IS primes serotonergic neurons in this region to be more sensitive to a subsequent heterotypic stressor. First, adult male rats were exposed to 100 min of intermittent IS to measure *tph2* mRNA expression 4 h after the onset of IS. In a second study, we assessed Tph2 protein expression 12 h and 24 h after the onset of IS. These time points were chosen based on a predicted time-delayed increase of Tph2 protein, compared to its mRNA, with respect to the natural diurnal variation of both *tph2* gene and Tph2 protein expression (Malek et al., 2004, Malek et al., 2007). In a third study, we exposed rats to IS followed by exposure to a heterotypic inescapable stressor 24 h later, a 10-min long exposure to cold swim stress at 15 °C, to address the effects on *tph2* mRNA expression. Cold swim stress was chosen because, similar to IS, forced swimming at this temperature also leads to escape deficits, passive stress coping, and increased anxiety-like behavior in rats (Christianson and Drugan, 2005).

## 2 Materials & methods

### 2.1 Animals

A total of 80 adult, male Sprague Dawley rats (Harlan Laboratories, Indianapolis, IN, USA; 200–250 g in weight) were used for our experiments. Rats were allowed to acclimate for one week, and were pair housed according to treatment group (home cage control, HC; inescapable tail shock, IS; cold swim stress, S) and/or euthanasia time point, respectively. Rats had *ad libitum* access to food (Cat. No. 8640; Teklad 22/5 Rodent Diet, Harlan Laboratories) and tap water under a regular 12:12 h light/dark cycle (lights on at 0600 h). Room temperature was maintained at 22 °C throughout the study. *Experiment 1* used 16 rats (*n* = 8 per group), while *Experiments 2* and *3* used 32 rats each (2 × 2 design, *n* = 8 per group). All animal procedures were approved by the University of Colorado Boulder Institutional Animal Care and Use Committee, and were compliant with the *Guide for the Care and Use of Laboratory Animals*, Eighth Edition (Institute for Laboratory Animal Research, The National Academies Press, Washington, D.C., 2011). All efforts were made to minimize the number of animals used and their suffering.

### 2.2 Experimental design

**Fig. 1:**
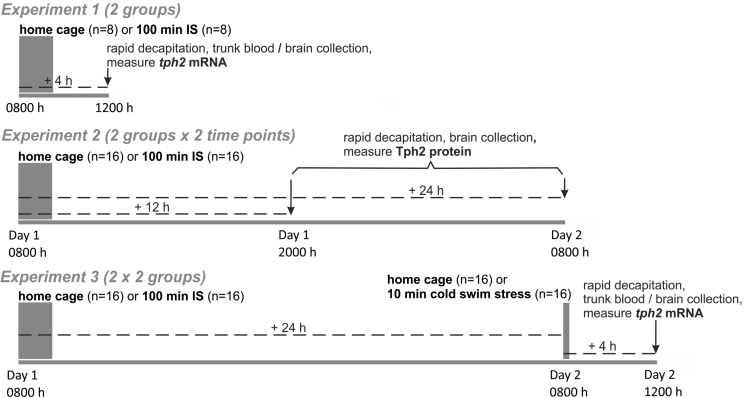
**Experimental design**. Three separate studies investigated the effects of inescapable tail shock (IS, duration of session: 100 min; number of tail shocks: 100, in random, unpredictable intervals with a 50–60 s inter-trial interval; intensity: 1.0 mA–1.6 mA) versus home cage (HC) control conditions on *tph2* mRNA and Tph2 protein expression in the dorsal raphe nucleus of adult male rats, and on the stressor-sensitivity of *tph2* mRNA expression following a second stressor (10-min session of cold swim stress at 15 °C) the next day. *Experiment 1* and *Experiment 2* addressed the time-dependent reactivity of *tph2* mRNA and Tph2 protein expression following 100 min of IS, respectively. *Experiment 3* addressed the interaction of IS on Day 1 and cold swim stress on Day 2 on *tph2* mRNA expression. In *Experiment 1,* rats were euthanized 4 h following the onset of IS (*n* = 8) versus HC (*n* = 8). In *Experiment 2,* rats were exposed to HC (*n* = 16) or IS (*n* = 16). Half of each treatment group was then euthanized 12 h following the onset of IS, while the other half was euthanized 24 h following the onset of IS. In *Experiment 3,* rats were exposed to HC (*n* = 16) or IS (*n* = 16) on Day 1, then to HC (*n* = 16) or cold swim stress (*n* = 16) on Day 2, and were euthanized 4 h following the onset of cold swim stress (28 h after the onset of IS). Abbreviations: IS, inescapable tail shock; *tph2*, tryptophan hydroxylase gene; Tph2, tryptophan hydroxylase 2 protein.

The experimental timelines are illustrated in Fig. 1. *Experiment 1* investigated whether one 100-min session of intermittent inescapable tail shock (IS, *n* = 8) was sufficient to alter *tph2* mRNA expression in subdivisions of the DR in comparison to home cage control rats (HC, *n* = 8). All rats were euthanized via rapid decapitation 4 h after the onset of IS; brains were dissected and fresh-frozen on dry ice, then stored at −80 °C, and later analyzed using *in situ* hybridization histochemistry (ISHH) for measurement of *tph2* mRNA expression. Trunk blood was collected on ice into 2 ml Eppendorf tubes containing 50 μl of 2% ethylenediaminetetraacetic acid (EDTA; Cat. No. E9884, Sigma-Aldrich, St. Louis, MO, USA) and 5% heparin (Cat. No. H4784, Sigma-Aldrich), centrifuged for 15 min at 3000 r.p.m., and stored at −80 °C, before processing in assays measuring plasma concentrations of corticosterone (CORT), interleukin (IL) 1β, IL-6, and IL-10.

In *Experiment 2* we measured Tph2 protein expression either 12 h (*n* = 8) or 24 h (*n* = 8) after the onset of one 100-min session of IS. Home cage control rats were also either euthanized at the 12 h time point (*n* = 8) or at the 24 h time point (*n* = 8). Rats were euthanized using rapid decapitation, and brains were dissected, fresh-frozen on dry ice, stored at −80 °C, and processed using western blot analysis for Tph2 protein.

*Experiment 3* addressed the question of whether IS sensitizes serotonergic neurons in the DR to a subsequent heterotypic stressor 24 h later, namely a 10-min exposure to cold swim stress (S) in 15 °C water (*n* = 8). *Experiment 3* was a 2 (*HC* versus *IS*) x 2 (*HC* versus *S*) design. We also measured plasma CORT, IL-1β, IL-6, and IL-10 concentrations to identify potential correlates of stress-induced changes in *tph2* mRNA expression. All animals were euthanized via rapid decapitation 4 h after the onset of S or home cage control conditions on Day 2; brains and trunk blood were processed as described above.

For details about the choice of euthanasia time points, the IS procedure, the cold swim stress, the behavioral analysis and the corticosterone and cytokine assays, please see supplemental materials.

### 2.3 Brain tissue sectioning and *in situ* hybridization histochemistry for *tph2* mRNA

After euthanasia, dissection of brain tissue, and temporary storage at −80 °C, hindbrains were separated from forebrains at the level of the mammillary bodies using a razor blade and a rat brain matrix (RBM-4000C, ASI Instruments, Warren, MI, USA), cryosectioned coronally at 12 μm in a series of 7 alternating sets of slides (84 μm anatomical distance between neighboring brain sections on any one slide) using a cryostat (Model CM 1900; Leica, Wetzlar, Germany) set to −20 °C chamber temperature, thaw-mounted onto Vista-Vision HistoBond^®^ microscope slides (VWR Scientific), and stored at −80 °C. *In situ* hybridization histochemistry (ISHH) for detection of *tph2* mRNA expression was performed using a cRNA riboprobe as previously described (Donner and Handa, 2009, Donner et al., 2012b). For details, see supplemental materials.

### 2.4 Semi-quantitative analysis of *tph2* mRNA expression

Digital autoradiography images of *tph2* mRNA expression in the DR, captured from the Kodak BioMax film, were analyzed with ImageJ (NIH, Bethesda, MD, USA) by an experimenter blinded to the treatment groups. To measure ‘gray value x area’ we used matrices in the shape of each subdivision of the brainstem DR (Donner et al., 2012a). Area (mm^2^) was defined as the area (within each matrix) that fell above a certain gray value threshold. This threshold was determined empirically and kept consistent throughout each experiment's analysis. Based on Gardner et al., 2009a, Gardner et al., 2009b, Abrams et al. (2004), and Donner et al. (2012a), a total of 13 to 14 rostro-caudal sections, designated levels +7 to −6, containing 7 major subdivisions of the DR were analyzed: rostral aspect of the dorsal raphe nucleus, dorsal part (rDRD), −7.580 to −7.916 mm bregma; rostral aspect of the dorsal raphe nucleus, ventral part (rDRV), −7.580 to −7.916 mm bregma; dorsomedial DR (caudal aspect of the dorsal raphe nucleus, dorsal part (cDRD)), −8.000 to −8.336 mm bregma; caudal aspect of the dorsal raphe nucleus, ventral part (cDRV), −8.000 to −8.504 mm bregma; dorsal raphe nucleus, ventrolateral part/ventrolateral periaqueductal gray (DRVL/VLPAG), −7.832 to −8.504 mm bregma; dorsal raphe nucleus, caudal part (DRC), −8.420 to −8.672 mm bregma; and dorsal raphe nucleus, interfascicular part (DRI), −8.420 to −8.672 mm bregma (Paxinos and Watson, 1998). Based on previous studies (Donner et al., 2012a), we subdivided the DRD and DRV into 5 rostral levels (rDRD and rDRV; designated analysis levels +7, +6, +5, +4, and +3; from −7.580 to −7.916 mm bregma) and 5 caudal levels (dorsomedial DR (cDRD); designated analysis levels +2, +1, 0, −1 and −2, from −8.000 to −8.336 mm bregma) or 7 caudal levels (cDRV; designated analysis levels +2, +1, 0, −1, −2, −3 and −4, from −8.000 to −8.504 mm bregma), respectively. This allowed for better functional interpretations because rostral and caudal aspects of the DRD and DRV are associated with different neuronal, behavioral and physiological responses to stressors (Abrams et al., 2005, Lowry et al., 2008, Hale and Lowry, 2011). An average value was computed for the DRVL/VLPAG using values from both the left and right hemisphere of each rat. Background was measured individually within each image, placing a circular matrix over adjacent gray matter, and was subtracted from each value. Final *tph2* mRNA expression values ((gray value above threshold – background) x area above threshold within matrix) for each anatomical subdivision were averaged, including an overall average value for the entire DR (all subdivisions combined).

### 2.5 Western blot analysis of Tph2 protein expression

After euthanasia, dissection of brain tissue, and temporary storage at −80 °C, hindbrains were separated from forebrains at the level of the mammillary bodies using a razor blade and a rat brain matrix (ASI Instruments, Warren, MI, USA), then cryosectioned coronally at 300 μm using a cryostat set to −10 °C chamber temperature. All brain sections were thaw-mounted onto glass microscope slides (Cat. No. 16004–420, VWR) as described previously (Evans et al., 2009). Sections were kept at −80 °C until microdissection at −10 °C, using a cold plate (Model No. TCP-2, Cat. No. Z176664, Sigma-Aldrich), and microdissections of 5 anatomical subdivisions of the DR (DRC, DRD, DRI, DRV, and DRVL/VLPAG, see above for abbreviations and Table 1 for details) were performed at −7.50 mm, −7.80 mm, −8.10 mm, −8.40 mm, and −8.70 mm from bregma, using blunt dissection needles ranging from 410 μm to 690 μm in inner diameter (Fine Science Tools, Foster City, CA, USA). Collection of rostral versus caudal aspects of the DRD or DRV was not feasible, given the 300 µm thickness of the brain sections. Rostrocaudal levels for microdissection were verified using a stereotaxic rat brain atlas (Paxinos and Watson, 1998), and all micropunches of a given anatomical DR subdivision (spanning 2 to 3 bregma levels) were pooled into 60 μl HEPES buffer (0.88% HEPES in ddH_2_O; Cat. No. 90909C, Sigma-Aldrich) containing 0.25% ‘Protease-Inhibitor Cocktail Set III’ (Cat. No. 539134, Millipore, Billerica, MA, USA) to be homogenized with a dispensable, sterile plastic pestle (Cat. No. K749520-0090, Fisher Scientific, Hampton, NH, USA). Samples were stored at −80 °C until western blot analysis. For details regarding the western blot procedures, refer to supplemental materials.

**Table 1:** Hindbrain microdissection for western blot analysis of Tph2 protein expression in *Experiment 2*.

Brain region (DR subdivision)	Distance from bregma	Inner diameter of microdissection needle	Total number of microdissections collected (pooled)
DRD	−7.50 mm	410 μm	3
	−7.80 mm	410 μm	
	−8.10 mm	410 μm	
DRV	−7.50 mm	410 μm	3
	−7.80 mm	410 μm	
	−8.10 mm	410 μm	
DRVL/VLPAG (left and right side)	−7.80 mm	410 μm (x 2)	4
	−8.10 mm	410 μm (x 2)	
DRC	−8.40 mm	690 μm	2
	−8.70 mm	690 μm	
DRI	−8.40 mm	410 μm	2
	−8.70 mm	410 μm	

For each brain region (DR subdivision), the total number of microdissections (2–4) were pooled to allow for proper signal detection. Abbreviations: DRC, dorsal raphe nucleus, caudal part; DRD, dorsal raphe nucleus, dorsal part; DRI, dorsal raphe nucleus, interfascicular part; DRV, dorsal raphe nucleus, ventral part; DRVL, dorsal raphe nucleus, ventrolateral part; VLPAG, ventrolateral periaqueductal gray.

### 2.6 Statistical analysis

Data were analyzed using SPSS (version 24.0, SPSS Inc., Chicago, IL, USA) after elimination of statistical outliers based on critical values (*z*-scores) of a two-tailed Grubbs' test (Grubbs, 1969) with α = 0.05. Based on these criteria, the following percentages of all values per measured parameter were excluded: 2.79% *tph2* mRNA expression (*Experiment 1*), 2.51% Tph2 protein expression, 0.81% *tph2* mRNA expression (*Experiment 3*), 0% cold swim behavior, 8.3% plasma corticosterone, 4.2% plasma IL-1β, 0% plasma IL-6, and 10.4% plasma IL-10. Overall and subdivision-specific *tph2* mRNA expression in *Experiments 1* and *3* were analyzed using a survey of linear mixed models (LMMs) with *IS* (*Experiments 1* and *3*: HC versus IS) and *cold swim* (*Experiment 3*: HC versus cold swim stress (S)) as the between-subjects factors, and *subdivision* and *rostrocaudal level* (7 separate DR subdivisions, namely the rDRV, cDRV, rDRD, dorsomedial DR (cDRD), DRVL/VLPAG, DRC and DRI, with 3–9 rostrocaudal levels each) as the within-subjects factors. Overall Tph2 protein expression in the entire DR was analyzed using LMM analysis with *time point* (12 h or 24 h time point) and *IS* (HC or IS) as the between-subjects factors, and *subdivision* (5 DR subdivisions; DRV, DRD, DRVL/VLPAG, DRC, DRI) as the within-subjects factor. For each LMM survey, various covariance structures were tested, and the best-fitting model (based on the −2 log-likelihood value) was chosen (Fox et al., 2017). Subdivision-specific Tph2 protein expression was analyzed with 2 (12 h or 24 h time point) x 2 (HC or IS) ANOVAs. Plasma cytokine and corticosterone concentrations were either compared using an individual Student's *t*-test (*Experiment 1*) or analyzed with multifactor analysis of variance (ANOVA) using *stress* (*Experiment 3*: HC versus IS on Day 1) and *swim* (*Experiment 3:* HC versus S on Day 2) as between-subjects factors. Where appropriate, LMMs or ANOVAs were followed by Fisher's least significant difference (LSD) *post hoc* tests (3 or more groups) or individual Student's *t*-tests for independent samples (2 groups) to determine pairwise differences between treatment groups. Furthermore, in the *in situ* hybridization histochemistry analysis, no *post hoc* analyses were conducted at specific levels of the rostrocaudal extent of subdivisions of the DR if one of the group sample sizes was below 50% of the full sample size for that treatment group. Additionally, *post hoc* analyses were conducted only when overall and secondary (i.e., within subdivision) LMMs yielded significant effects of *IS*, *cold swim* (*S*), *IS* × *S* interactions, or interactions among these factors and rostrocaudal level within raphe subdivision or rostrocaudal level. The percent time the rats spent climbing, swimming or immobile, and the number of dives during the cold swim procedure (*Experiment 3*) were compared using individual Student's *t*-tests (HC/S vs. IS/S). Graphs and figures were prepared using SigmaPlot (Systat Software Inc., San Jose, CA, USA) and CorelDraw (Version 12.0, Corel Inc., Mountain View, CA, USA). Significance was accepted at *p* < .05. Data are either shown as the mean + or ± the standard error of the mean (SEM), or as individual data points when visualizing correlations.

## 3 Results

### 3.1 Inescapable tail shock (IS) increases *tph2* mRNA expression in the dorsomedial DR (cDRD) 4 h later

**Fig. 2:**
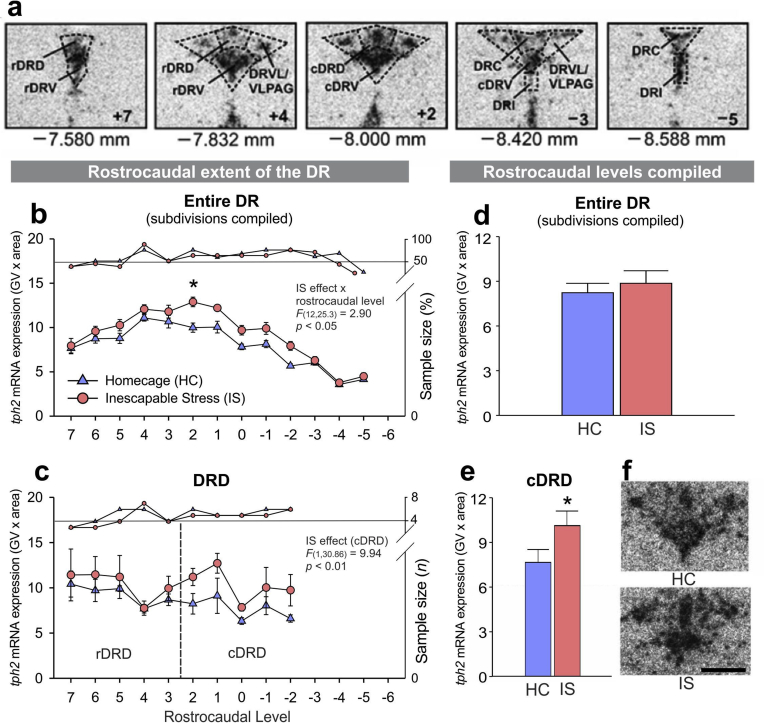
**Effects of inescapable tail shock (IS) on *tph2* mRNA expression**. (**a**) Representative bregma levels of the neuroanatomical analysis atlas for *tph2* mRNA expression in all anatomical subdivisions of the serotonergic dorsal raphe nucleus (DR) studied in an adult, male rat brain. (**b**) Mean ± SEM *tph2* mRNA expression throughout the rostrocaudal extent of the entire DR (subdivisions averaged), in *Experiment 1*. (**c**) Mean ± SEM *tph2* mRNA expression throughout the rostrocaudal extent of the dorsal raphe nucleus, dorsal part (DRD), including the rostral aspect (rDRD) and the caudal aspect (dorsomedial DR, cDRD) in *Experiment 1.* (**d**) Overall *tph2* mRNA expression in the entire DR (subdivisions and rostrocaudal levels averaged) after exposure to home cage control conditions (HC) or IS. All rats were euthanized 4 h after the onset of IS (IS, *n* = 8), including unstressed control rats (HC, *n* = 8). (**e**) Overall *tph2* mRNA expression in the dorsomedial DR (cDRD; rostrocaudal levels averaged). (**f**) Representative photomicrographs of *tph2* mRNA expression at bregma level–8.000 mm, measured using *in situ* hybridization histochemistry. *Post hoc* comparisons were made using Student's *t*-tests. *p < .05, IS vs HC. *Post hoc* testing was not conducted at a specific rostrocaudal level (7 through −6) when 1 or more groups contained less than half of the full sample size, indicated by the right y-axis. Rostrocaudal levels 7 = −7.580 mm, 6 = −7.664 mm, 5 = −7.748 mm, 4 = −7.832 mm, 3 = −7.916 mm, 2 = −8.000 mm, 1 = −8.084 mm, 0 = −8.168 mm, −1 = −8.252 mm, −2 = −8.336 mm, −3 = −8.420 mm, −4 = −8.504 mm, −5 = −8.588 mm, −6 = −8.672 mm from bregma. Scale bar: 1 mm.

In *Experiment 1*, analysis of IS-induced increases in *tph2* mRNA expression throughout the rostrocaudal extent of each subdivision of the entire DR using LMM revealed that IS-induced increases in *tph2* mRNA expression 4 h after the onset of IS were dependent on rostrocaudal level (Fig. 2b; *IS x rostrocaudal level* interaction, *F*_(12, 25.3)_ = 2.90, *p* < .05). Further analysis using secondary LMMs within subdivisions of the DR revealed that the overall IS effect was mainly due to increased *tph2* mRNA expression in the dorsomedial DR (cDRD; Fig. 2c and e; Table 2; *IS effect, F*_(1, 30.9)_ = 9.94, *p* < .01). No other subdivision of the DR, other than the dorsomedial DR (cDRD), displayed altered *tph2* mRNA expression in response to IS. For more details, please see Supplemental Table 1 and Supplemental Fig. 1.

**Table 2:** *tph2* mRNA and Tph2 protein expression in all subdivisions of the dorsal raphe nucleus (DR).

*Experiment 1*	*tph2* mRNA (*n* = 8 per group unless indicated otherwise)	
	Home cage	Inescapable tail shock
rDRD	8.92 ± 0.34 ^(*n*^^=^^7)^	9.36 ± 0.88
cDRD	7.27 ± 0.52 ^(*n*^^=^^6)^	10.21 ± 0.97 *
rDRV	11.41 ± 1.50 ^(*n*^^=^^7)^	11.13 ± 0.84
cDRV	8.73 ± 0.79 ^(*n*^^=^^7)^	11.12 ± 1.10
DRVL/VLPAG	6.83 ± 0.64	7.33 ± 0.41
DRC	5.51 ± 0.48	5.00 ± 0.61
DRI	6.06 ± 0.63	5.67 ± 0.75
Entire DR	8.29 ± 0.51	8.70 ± 0.63

*Experiment 2*	Tph2 protein (*n* = 8 per group unless indicated otherwise)			
	Home cage/12 h	Inescapable tail shock/12 h	Home cage/24 h	Inescapable tail shock/24 h
DRD	0.78 ± 0.22	1.22 ± 0.28	0.24 ± 0.10 ^#^	0.66 ± 0.15 *
DRV	0.89 ± 0.24	0.98 ± 0.19 ^(*n*^^=^^7)^	0.63 ± 0.08 ^(*n*=7)^	0.87 ± 0.14
DRVL/VLPAG	0.22 ± 0.07	0.33 ± 0.06	0.26 ± 0.05	0.31 ± 0.07
DRC	0.35 ± 0.03	0.31 ± 0.09 ^(*n*^^=^^7)^	0.16 ± 0.07 ^#^	0.22 ± 0.08 ^(*n*^^=^^6)^
DRI	0.24 ± 0.08	0.29 ± 0.04	0.18 ± 0.03	0.23 ± 0.03
Entire DR	0.48 ± 0.05	0.71 ± 0.10 *	0.32 ± 0.05 ^#^	0.49 ± 0.04 ^#,^ *

*Experiment 3*	*tph2* mRNA (*n* = 8 per group unless indicated otherwise)			
	Home cage/Home cage	Home cage/Swim	Inescapable tail shock/Home cage	Inescapable tail shock/Swim
rDRD	6.50 ± 0.44	6.89 ± 0.54	6.70 ± 0.62	7.54 ± 0.38
cDRD	7.37 ± 0.99	8.59 ± 0.86	9.24 ± 0.37	10.91 ± 0.31 ^#^
rDRV	9.47 ± 0.60	10.66 ± 0.83	10.21 ± 0.94	11.17 ± 0.64
cDRV	10.28 ± 0.84	10.90 ± 0.85	11.89 ± 0.61	12.09 ± 0.26 ^(*n*^^=^^7)^
DRVL/VLPAG	5.03 ± 0.65	5.50 ± 0.43	5.77 ± 0.23	6.21 ± 0.40
DRC	7.41 ± 0.45 ^(*n*^^=^^7)^	7.35 ± 0.43 ^(*n*^^=^^7)^	7.34 ± 0.99	7.11 ± 0.77
DRI	6.60 ± 0.27	6.39 ± 0.56	7.08 ± 0.56	7.44 ± 0.48
Entire DR	7.41 ± 0.60	7.69 ± 0.32 ^(*n*^^=^^7)^	8.32 ± 0.51	8.84 ± 0.35

*Experiment 1*: *p < .05 vs. the unstressed home cage control group (individual Student's *t*-test). *Experiment 2:* *p < .05 vs. the home cage control group at the same time point (IS effect); ^#^*p* < .05 vs. the 12 h time point of the same treatment group (diurnal rhythm effect). *Experiment 3*: ^#^*p* < .05 vs. IS/swim vs. the home cage/swim group (Fisher's LSD *post hoc* tests). Abbreviations: cDRD, dorsomedial DR (caudal aspect of the dorsal raphe nucleus, dorsal part); cDRV, caudal aspect of the dorsal raphe nucleus, ventral part; DRC, dorsal raphe nucleus, caudal part; DRI, dorsal raphe nucleus, interfascicular part; DRVL, dorsal raphe nucleus, ventrolateral part; rDRD, rostral aspect of the dorsal raphe nucleus, dorsal part; rDRV, rostral aspect of the dorsal raphe nucleus, ventral part; VLPAG, ventrolateral periaqueductal gray. Values are shown as the mean ± the standard error of the mean (SEM). Group sizes vary due to Grubbs' test-based outlier removal.

### 3.2 Inescapable tail shock (IS) increases Tph2 protein in the DRD 24 h later

**Fig. 3:**
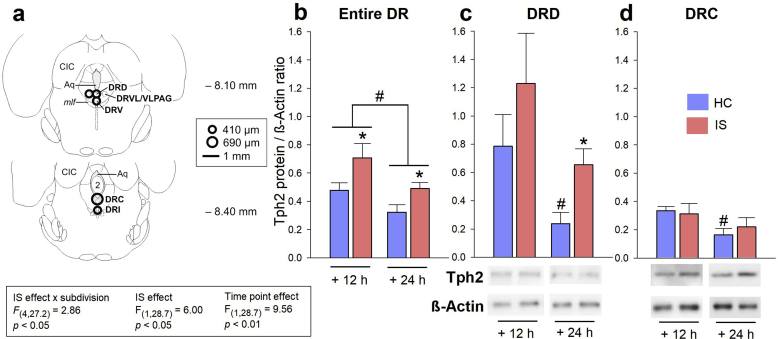
**Effects of inescapable tail shock (IS) on Tph2 protein expression 12 h and 24 h after IS**. (**a**) Representative rostrocaudal bregma levels of the microdissection atlas used to sample anatomical subdivisions of the dorsal raphe nucleus (DR) in the rat brainstem for *Experiment 2*. (**b**, **c**, and **d**) Tryptophan hydroxylase 2 (Tph2) protein expression in (**b**) the entire DR (subdivisions averaged), (**c**) the dorsal part of the dorsal raphe nucleus (DRD), and (**d**) the caudal part of the dorsal raphe nucleus (DRC) either 12 h or 24 h after the onset of IS, compared to home cage (HC) control conditions (HC/12 h, *n* = 8; IS/12 h, *n* = 8; HC/24 h, *n* = 8; IS/24 h, *n* = 8). Below (**c**) and (**d**), representative, inverted chemoluminescence photomicrographs of significant IS- or diurnal rhythm-induced changes of Tph2 protein expression. Beta-actin (β-Actin) was used as the loading control during the western blot assay. Abbreviations: 2, second cerebellar lobule; Aq, mesencephalic aqueduct; CIC, central nucleus of the inferior colliculus; DRC, dorsal raphe nucleus, caudal part; DRD, dorsal raphe nucleus, dorsal part; DRI, dorsal raphe nucleus, interfascicular part; DRV, dorsal raphe nucleus, ventral part; DRVL, dorsal raphe nucleus, ventrolateral part; *mlf*, medial longitudinal fasciculus; VLPAG, ventrolateral periaqueductal gray. *p < .05 vs. the HC control group of the same time point (IS effect); ^#^*p* < .05 vs. the 12 h time point of the same treatment group. Pairwise *post hoc* comparisons were performed using the Student's *t*-test for independent samples.

Analysis of Tph2 protein expression in *Experiment 2* (applying the LMM model, using measurements of all subdivisions of the DR; rostrocaudal levels of each subdivision had to be pooled for each rat to enable signal detection) revealed an interaction between *IS* x *subdivision* (Fig. 3a and Fig. 3b; *IS effect* x *subdivision, F*_(4, 27.2)_ = 2.86, *p* < .05), as well as main effects of IS and time point (Fig. 3a and b; *IS effect*, *F*_(1, 28.7)_ = 6.00, *p* < .05; *time point effect, F*_(1, 28.7)_ = 9.56, *p* < .01). Average Tph2 protein expression in the entire DR (subdivisions compiled) was increased 12 h (measured at 2000 h, 2 h after lights off) and 24 h (measured at 0800 h, 2 h after lights on) following IS, compared to unstressed controls at the same time point (Fig. 3b, *p* < .05), and all rats expressed less Tph2 protein at the 24 h time point compared to the 12 h time point (Fig. 3b, *p* < .05). In the DRD, Tph2 protein expression remained unaffected 12 h following the onset of IS, compared to HC controls at the same time point, but was increased 24 h (*p* < .05) following the onset of IS, compared to HC controls at the 24 h time point (Fig. 3c; Table 2; *IS effect*, *F*_(1, 28)_ = 4.61, *p* < .05). In addition, a diurnal rhythm of Tph2 protein expression was detected in the DRD (Fig. 3c; Table 2; *time point effect*, *F*_(1, 28)_ = 7.58, *p* < .01) and in tendency also in the DRC (Fig. 3d; *time point effect*, *F*_(1, 25)_ = 4.02, *p* = .06). *Post hoc* comparisons demonstrated that the diurnal rhythm effect was only present in the DRD and DRC of HC control rats (*p* < .05), but not in the DRD or DRC of rats that were exposed to IS. No other DR subdivisions displayed an IS effect or a diurnal rhythm for Tph2 protein expression. As expected, overall Tph2 protein expression differed significantly depending on the DR subdivision (Supplemental Table 2; *DR subdivision effect*; *F*_(4, 27.2)_ = 21.87, *p* < .001). For more details, please see Supplemental Table 2 and Supplemental Fig. 2.

### 3.3 Inescapable tail shock (IS) and cold swim independently increase tph2 mRNA expression in the caudal DRD 28 h after IS (4 h after cold swim)

**Fig. 4:**
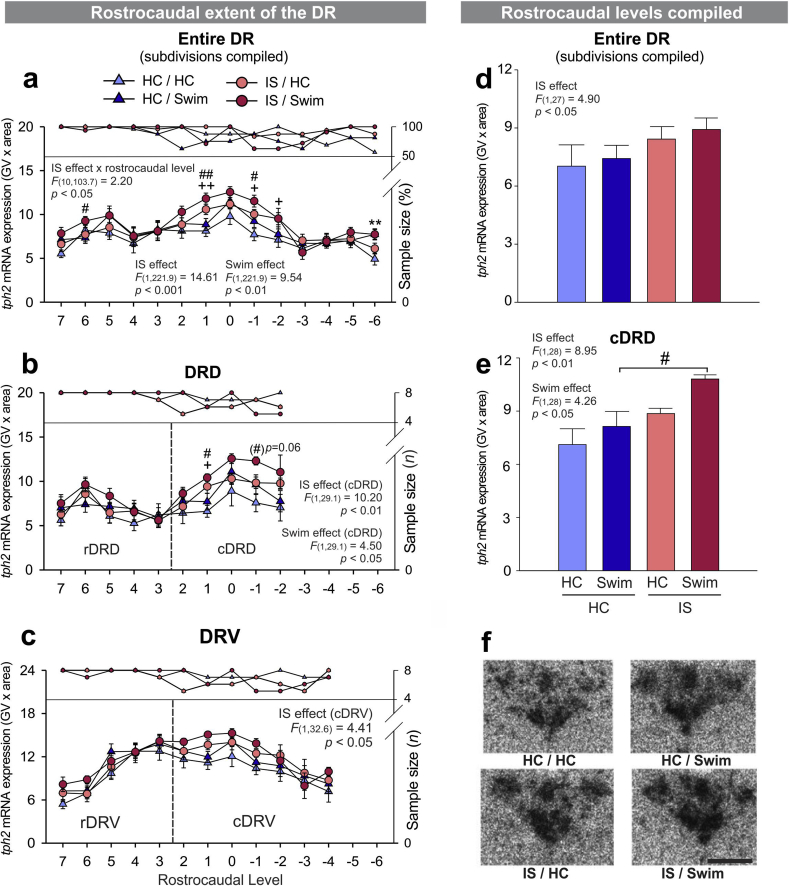
**Effects of inescapable tail shock (IS) and cold swim on *tph2* mRNA expression**. (**a**–**c**) Mean ± SEM of *tph2* mRNA expression throughout the rostrocaudal extent of (**a**) the entire DR (subdivisions averaged), (**b**) the dorsal raphe nucleus, dorsal part (DRD), including the rostral aspect (rDRD) and the caudal aspect (dorsomedial DR, cDRD), and (**c**) the dorsal raphe nucleus, ventral part, including the rostral aspect (rDRV) and the caudal aspect (cDRV), in *Experiment 3*. (**d**–**e**) Overall *tph2* mRNA expression in (**d**) the entire DR (subdivisions and rostrocaudal levels averaged) and in (**e**) the dorsomedial DR (cDRD; rostrocaudal levels averaged). All rats were euthanized 4 h after the onset of cold swim stress on Day 2, including unstressed control rats (HC/HC, *n* = 8), rats that remained in their home cage on Day 1 and were exposed to cold swim on Day 2 (HC/Swim, *n* = 8), rats that received IS on Day 1 and stayed in the home cage on Day 2 (IS/HC, *n* = 8), and rats that were stressed with IS on Day 1 and with cold swim on Day 2 (IS/Swim, *n* = 8). (**f**) representative photomicrographs of *tph2* mRNA expression in all four treatment groups at bregma level −8.000 mm, measured via *in situ* hybridization histochemistry. *Post hoc* comparisons were performed using Fisher's Least Significant Difference (LSD) tests. ^**^p < .01, HC/HC versus HC/Swim; ^+^*p* < .05 and ^++^*p* < .01, HC/HC versus IS/HC; ^#^*p* < .05, ^##^*p* < .01, HC/Swim versus IS/Swim. *Post hoc* testing was not conducted at a specific rostrocaudal level (7 through −6) when 1 or more groups contained less than half of the full sample size, indicated by the right y-axis. Rostrocaudal levels 7 = −7.580 mm, 6 = −7.664 mm, 5 = −7.748 mm, 4 = −7.832 mm, 3 = −7.916 mm, 2 = −8.000 mm, 1 = −8.084 mm, 0 = −8.168 mm, −1 = −8.252 mm, −2 = −8.336 mm, −3 = −8.420 mm, −4 = −8.504 mm, −5 = −8.588 mm, −6 = −8.672 mm from bregma. Scale bar: 1 mm.

Analysis of overall *tph2* mRNA expression in *Experiment 3* (applying the LMM model, using measurements at all rostrocaudal levels of all subdivisions of the DR) revealed an interaction between *IS* x *rostrocaudal level* (Fig. 4a; *IS effect* x *rostrocaudal level,* *F*_(10, 103.7)_ = 2.20, *p* < .05), as well as main effects of *IS* (Fig. 4a; *F*_(1, 221.9)_ = 14.61, *p* < .001) and *cold swim* (Fig. 4a; *F*_(1, 221.9)_ = 9.54, *p* < .01). An ANOVA of the average *tph2* mRNA expression in the entire DR (rostrocaudal levels and subdivisions compiled) also detected a main effect of *IS* (Fig. 4d; *F*_(1, 27)_ = 4.90, *p* < .05). Subdivision-specific LMM analysis of *tph2* mRNA expression, using data points from each rostrocaudal level, revealed effects of both IS and cold swim in the dorsomedial DR (cDRD; Fig. 4b; Table 2, *IS effect, F*_(1, 29.1)_ = 10.20, *p* < .01; *cold swim effect, F*_(1, 29.1)_ = 4.50, *p* < .05). When all rostrocaudal levels of the dorsomedial DR (cDRD) were averaged, *post hoc* pairwise comparisons showed that IS/S rats expressed significantly more *tph2* mRNA in this subdivision than their HC/S counterparts (Fig. 4e; Table 2; Supplemental Fig. 3g, *p* < .05). The rostrocaudal extent of the cDRV also displayed increased *tph2* mRNA due to IS (Fig. 4c; Supplemental Fig. 3b; *IS effect, F*_(1, 32.6)_ = 4.41, *p* < .05), but *post hoc* pairwise comparisons of the average *tph2* mRNA expression in the entire cDRV were not significant (Supplemental Fig. 3i). No other subdivision showed a significant main effect of IS or cold swim. LMM analysis of the rostrocaudal extent of the DRVL/VLPAG, however, revealed a significant *IS* x *rostrocaudal level* interaction (Supplemental Fig. 3c; *F*_(8, 14.6)_ = 7.70, *p* < .001), but *post hoc* comparisons of the average *tph2* mRNA expression in the entire DRVL/VLPAG were not significant (Supplemental Fig. 3j). In contrast, c*old swim x rostrocaudal level* interactions were found in both the DRC (Supplemental Fig. 3d; *F*_(3, 25.1)_ = 3.66, *p* < .05) and the DRI (Supplemental Fig. 3e; *F*_(3, 26.8)_ = 10.68, *p* < .001), again without significant *post hoc* comparisons of the average *tph2* mRNA expression in respective subdivisions (Supplemental Figs. 3k and 3l). For more details, please refer to Supplemental Table 3 and Supplemental Fig. 3.

### 3.4 Inescapable tail shock (IS)-induced immobility during cold swim

**Fig. 5:**
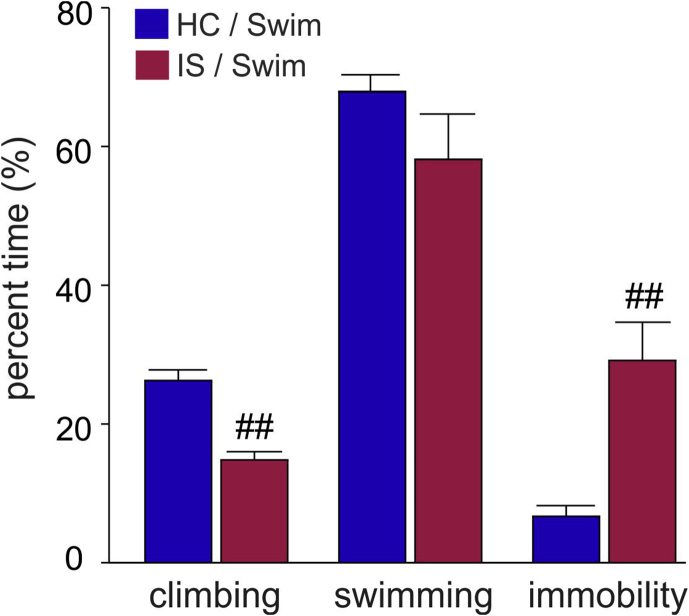
**Behavior during cold swim stress**. Behavior of rats during the 10-min cold swim procedure in *Experiment 3.* Rats were exposed to home cage control conditions (HC, *N* = 16) or inescapable tail shock (IS, *N* = 16) on Day 1. On Day 2, half of the rats of each treatment group were exposed to cold swim for 10 min in 15 °C water (HC/Swim, *n* = 8; IS/Swim, *n* = 8) while the other half remained in their home cages. The percent time spent climbing, swimming or immobile is shown. No difference was detected in the number of dives. ^##^*p* < .01 vs. HC/Swim group (Student's *t*-test for independent samples).

During the 10-min cold swim (S) procedure, rats that had been exposed to IS the day before, relative to home cage controls, spent less percent time climbing (*p* < .01), and more percent time immobile (*p* < .01), while IS had no effect on the percent time spent swimming or the number of dives (Fig. 5).

### 3.5 Increased plasma corticosterone 4 h after cold swim and 28 h after inescapable tail shock (IS)

Plasma corticosterone was found to be elevated 4 h after the onset of S exposure on Day 2 in *Experiment 3* (Table 3), but not 4 h after the onset of IS in *Experiment 1* (Table 3). Cold swim stress thus appears to increase plasma corticosterone for up to 4 h, while plasma corticosterone of IS-exposed rats appears to return to HC control levels within this 4 h time frame, as previously reported (Fleshner et al., 1993). Interestingly, IS resulted in elevated plasma corticosterone concentrations in IS/HC rats 28 h after the onset of IS, compared to HC/HC control rats (Table 3; *IS effect, F*_(1,27)_ = 9.73, *p* < .01; *cold swim effect, F*_(1, 27)_ = 3.63, *p* = .07; *IS effect* x *cold swim effect, F*_(1, 25)_ = 3.37, *p* = .08), also consistent with previous studies (Fleshner et al., 1993).

**Table 3:** Plasma concentrations of corticosterone and cytokines IL-1β, IL-6 and IL-10 (*n* = 8 per group unless indicated otherwise).

*Experiment 1*	Home cage	Inescapable tail shock
Corticosterone (ng/ml)	36.55 ± 9.91	57.06 ± 15.02 ^(*n* = 7)^
IL-1β (pg/ml)	21.76 ± 14.04 ^(*n* = 7)^	20.69 ± 9.47
IL-6 (pg/ml)	72.25 ± 14.04	91.63 ± 10.86
IL-10 (pg/ml)	4.45 ± 2.97 ^(*n* = 7)^	17.82 ± 8.69 ^(*n* = 7)^

*Experiment 3*	Home cage/Home cage	Home cage/Swim	Inescapable tail shock/Home cage	Inescapable tail shock/Swim
Corticosterone (ng/ml)	40.87 ± 7.32 ^(*n* = 7)^	83.76 ± 16.41*	97.67 ± 8.60 ^(*n* = 7)^ **	98.46 ± 8.84 ^(*n* = 7)^
IL-1β (pg/ml)	61.24 ± 26.83 ^(*n* = 7)^	30.72 ± 24.22	104.59 ± 35.80	87.91 ± 31.38
IL-6 (pg/ml)	77.21 ± 3.26	83.37 ± 4.90	92.32 ± 8.98	107.46 ± 9.10^#^
IL-10 (pg/ml)	22.95 ± 10.37 ^(*n* = 7)^	34.21 ± 12.51	53.17 ± 20.37 ^(*n* = 7)^	10.18 ± 5.66 ^(*n* = 7)#, &&^

*Experiment 3*: *p < .05 and **p < .01 vs. the home cage/home cage group; ^#^*p* < .05 vs. the home cage/swim group; ^&^*p* < .05 and ^&&^*p* < .01 vs. the inescapable tail shock/home cage group (Fisher's LSD *post hoc* tests). Values are shown as the mean ± the standard error of the mean (SEM). Group sizes vary due to Grubbs' test-based outlier removal.

### 3.6 Proinflammatory cytokine milieu after inescapable tail shock (IS) followed by cold swim

Inescapable tail shock by itself was not sufficient to alter plasma concentrations of IL-1β, IL-6, or IL-10 when measured 4 h after the onset of IS (for details see Table 3). However, rats that were exposed to both stressors (IS/S group) displayed a significant elevation of plasma IL-6, compared to HC/S rats (*p* < .05) (Table 3; *IS effect, F*_(1, 30)_ = 7.75, *p* < .05; *cold swim effect, F*_(1, 30)_ = 2.29, *p* = .14; *IS effect* x *cold swim effect, F*_(1, 28)_ = 0.41, *p* = .53). An IS × S interaction also was observed for plasma IL-10, resulting in a significant decrease of plasma IL-10 in rats that were exposed to both stressors (IS/S group), as measured 4 h after the onset of S, compared to HC/S (*p* < .05) and IS/HC rats (*p* < .01; Table 3; *IS effect, F*_(1, 27)_ = 0.05, *p* = .81; *cold swim effect, F*_(1, 27)_ = 1.42, *p* = .25; *IS effect* x *cold swim* interaction, *F*_(1, 25)_ = 4.53, *p* = .05). At the given time points (4h or 28h after the onset of IS, or 4h after S, respectively), no significant effects of *IS*, *cold swim* or an interaction between the two stressors were detected on the concentration of plasma IL-1ß. For correlations between *tph2* mRNA expression and cold swim behavior, and *tph2* expression and cytokine concentrations, see supplemental materials.

## 4 Discussion

Exposure to one 100 min-long session of IS was sufficient to increase *tph2* mRNA expression in the dorsomedial DR (caudal aspect of the dorsal DR, cDRD), as measured 4 h after the onset of IS; furthermore, rats that were exposed to IS on Day 1 also responded with increased *tph2* mRNA in the dorsomedial DR (cDRD) 4 h after exposure to the heterotypic cold swim stressor on Day 2, relative to rats maintained in the home cage on Day 1 and exposed to cold swim on Day 2. Consistent with these findings, IS also increased Tph2 protein expression in the DRD (rostral and caudal aspects combined), as measured 24 h after the onset of IS. Furthermore, among rats exposed to home cage control conditions on Day 1, *tph2* mRNA expression in the dorsomedial DR (cDRD) was inversely correlated with climbing behavior during forced swimming, suggesting that individual variability in serotonergic signaling in the dorsomedial DR (cDRD) may be relevant to depressive-like, passive behavioral responses. Combined exposure to IS and swim stress resulted in a proinflammatory cytokine milieu (increased IL-6, decreased IL-10). Finally, among rats exposed to home cage control conditions on Day 1, *tph2* mRNA expression in the dorsomedial DR (cDRD) was positively correlated with plasma IL-6 concentrations.

For detailed descriptions of the neuroanatomical organization and different functionalities of subdivisions of the DR and MnR (Lowry, 2002, Lowry et al., 2005, Lowry et al., 2008, Lowry and Hale, 2010, Hale and Lowry, 2011) and differing human (Baker et al., 1990, Baker et al., 1991) versus rodent nomenclature (Dahlstroem and Fuxe, 1964, Paxinos and Watson, 1998, Franklin and Paxinos, 2008), Michelsen et al. (2007) provides a comprehensive historical and functional review. In comparison to our nomenclature, Muzerelle et al. (2016), for example, implements the nomenclature originally used for the rat serotonergic system (B5-B9 cell groups (see Dahlstroem and Fuxe, 1964)). Accordingly, B5 serotonergic neurons are located in the DRI, B6 serotonergic neurons in the DRC, B7 serotonergic neurons in the DRD (B7d), DRV (B7v), and DRVL/VLPAG (B7l), B8 serotonergic neurons in the median raphe nucleus (MnR), and B9 serotonergic neurons are scattered throughout the supralemniscal and pontomesencephalic reticular formation lateral to the MnR. According to previously published work from our group (Donner et al., 2012a), we further subdivided the B7d region (DRD) into rostral and caudal subregions (rDRD, cDRD) and the B7v region (DRV) into rostral and caudal subregions (rDRV, cDRV). Refer to Table 4 for an overview of terminology used in the selected, representative literature. In line with work by Christianson et al., 2010, Commons, 2008, Lowry et al. (2008) and Spiacci et al. (2016), the cDRD is equivalent to the middle dorsal DR or “dorsomedial DR”, a unique stress-responsive region that projects to stress-related structures within the limbic system (Commons et al., 2003, Abrams et al., 2005, Lowry et al., 2008, Lowry and Hale, 2010, Hassell et al., 2017), as described in more detail in the following paragraph.

**Table 4:** Neuroanatomical nomenclature of serotonergic systems in the rodent hindbrain.

Serotonergic regions and subdivisions	References	B5–B9 terminology used by other authors	References	Other commonly accepted terminology	References	Distance from bregma in the rat brain
**DRD**	Paxinos and Watson, 1998	B7d	Muzerelle et al., 2016			−7.580 to −8.336 mm
rDRD	Donner et al., 2012a			rostral dorsal DR	Commons 2008	−7.580 to −7.916 mm
cDRD	Donner et al., 2012a			middle dorsal DR, dorsomedial DR	Commons et al., 2003, Commons, 2008, Lowry et al., 2008, Okere and Waterhouse, 2006, McEuen et al., 2008, Spiacci et al., 2016	−8.000 to −8.336 mm
DRD shell vs. DRD core	Abrams et al., 2005, Hioki et al., 2010					−7.580 to −8.336 mm
**DRV**	Paxinos and Watson, 1998	B7v	Muzerelle et al., 2016			−7.580 to −8.504 mm
rDRV	Donner et al., 2012a			rostral ventral DR	Commons 2008	−7.580 to −7.916 mm
cDRV	Donner et al., 2012a			middle ventral DR	Commons 2008	−8.000 to −8.504 mm
**DRVL/VLPAG** (left and right side)	Paxinos and Watson, 1998	B7l	Muzerelle et al., 2016	lateral wings	Commons 2008	−7.832 to −8.504 mm
**DRC**	Paxinos and Watson, 1998	B6	Jensen et al., 2008, Okaty et al., 2015, Muzerelle et al., 2016	caudal dorsal DR	Commons 2008	−8.420 to −8.672 mm
**DRI**	Paxinos and Watson, 1998	B5	Jensen et al., 2008, Okaty et al., 2015, Muzerelle et al., 2016	caudal ventral DR	Commons 2008	−8.420 to −8.672 mm
**MnR**	Paxinos and Watson, 1998	B8	Jensen et al., 2008, Okaty et al., 2015, Muzerelle et al., 2016			−7.832 to −8.504 mm
rMnR	Fox et al., 2017					−7.832 to −8.084 mm
cMnR	Fox et al., 2017					−8.168 to −8.504 mm
**B9/PMRF**	Fox et al., 2017, Paxinos and Watson, 1998, Vertes and Crane, 1997	B9	Jensen et al., 2008, Okaty et al., 2015, Muzerelle et al., 2016			−7.580 to −9.260 mm

Abbreviations: cDRD, dorsomedial DR (caudal aspect of the dorsal raphe nucleus, dorsal part); cDRV, caudal aspect of the dorsal raphe nucleus, ventral part; cMnR, caudal aspect of the median raphe nucleus; DRC, dorsal raphe nucleus, caudal part; DRD, dorsal raphe nucleus, dorsal part; DRI, dorsal raphe nucleus, interfascicular part; DRV, dorsal raphe nucleus, ventral part; DRVL, dorsal raphe nucleus, ventrolateral part; MnR, median raphe nucleus; PMRF, pontomesencephalic reticular formation; rDRD, rostral aspect of the dorsal raphe nucleus, dorsal part; rDRV, rostral aspect of the dorsal raphe nucleus, ventral part; rMnR, rostral aspect of the median raphe nucleus; VLPAG, ventrolateral periaqueductal gray.

Expression of *tph2* mRNA was elevated in the dorsomedial DR (cDRD) 4 h after the onset of IS. A multitude of studies have identified the DRD, particularly the dorsomedial DR (cDRD), as an important stress- and anxiety-related subdivision of the DR (Hammack et al., 2002, Commons et al., 2003, Lowry and Hale, 2010, Rozeske et al., 2011, Hassell et al., 2017). Collateral efferent projections from this region branch out to innervate anxiety-and stress-related limbic and cortical structures, including the medial prefrontal cortex, the basolateral amygdala (BLA), the bed nucleus of the stria terminalis (BNST), the central amygdaloid nucleus (CeA) and the periventricular hypothalamus (for review see Lowry et al., 2005, Lowry et al., 2008, Hale and Lowry, 2011). Furthermore, serotonergic neurons in this region are activated by various anxiogenic drugs, including caffeine, N-methyl-β-carboline-3-carboximide (FG-7142), and m-chlorophenylpiperazine (mCPP), a 5-HT_2C_ receptor agonist (Abrams et al., 2005), and the anxiety-related peptide urocortin 2 (Amat et al., 2004, Hale et al., 2010). Exposure to IS, an open-field arena, or social defeat also activates DRD serotonergic neurons (Amat et al., 2005, Gardner et al., 2005, Bouwknecht et al., 2007), and chronic systemic corticosterone treatment specifically sensitizes the DRD to acoustic startle-induced increases in Tph2 activity, measured as the stress-induced accumulation of 5-hydroxytryptophan (5-HTP) following systemic blockade of the enzymatic conversion of 5-HTP to 5-HT (Donner et al., 2016). Stress-induced activation of the DRD may be driven by corticotropin-releasing factor (CRF) receptor type 2 (CRFR2)-mediated afferent signaling from the BNST (Sink et al., 2013). The DRD contains serotonergic neurons that co-express CRF (Commons et al., 2003), and has been shown to express *crfr2* mRNA within serotonergic neurons (Day et al., 2004). Moreover, it has the highest density of CRFR2-immunoreactive serotonergic neurons (Lukkes et al., 2011), and local microinjections of the highly CRFR2-selective agonist urocortin 2 into the dorso-caudal DR mimic the behavioral consequences of IS (in the absence of IS). In addition, blockade of CRFR2 via a locally administered antagonist prevents the behavioral manifestations of IS (Hammack et al., 2003). Most strikingly, overexpression of CRF in the BNST alters CRFR2 density specifically within the DRD and DRC (Sink et al., 2013). Thus, IS may not only stimulate the immediate release of serotonin within the DR (Maswood et al., 1998), but may also lead to a BNST-driven, CRFR2-mediated increase of new serotonin synthesis via elevated *tph2* expression in the dorsomedial DR (cDRD). This hypothesis is supported by findings that the BNST is necessary for the effects of IS (Hammack et al., 2004). Nevertheless, the subdivision-specific effects of IS on *tph2* mRNA expression, measured 4 h following the onset of IS, must be interpreted with caution because our overall LMM analysis detected an interaction of *IS x rostrocaudal level*, but no interaction of *IS x subdivision*.

While both IS and cold swim stress independently elevated overall *tph2* mRNA expression, subdivision-dependent effects were again restricted to the dorsomedial DR (cDRD). Rats that were exposed to IS on Day 1 responded with increased *tph2* mRNA in the dorsomedial DR (cDRD) upon exposure to the heterotypic cold swim stress on Day 2, while rats exposed to HC control conditions on Day 1 did not. This, again, indicates the unique stress-related sensitivity of this region. The mechanism underlying this sensitivity may be due to an IS-induced desensitization of autoinhibitory 5-HT_1A_ receptors (Rozeske et al., 2011), which is likely to make the dorsomedial DR (cDRD) more vulnerable to a subsequent, heterotypic stress exposure 24 h following IS. The behavioral effects of IS are known to last for at least 24 h. For example, IS reduces juvenile social exploration 24 h later, and this is accompanied by increased 5-HT release within the BLA in response to the juvenile exploration testing (Christianson et al., 2010). The BLA is a major target of serotonergic neurons from the dorsomedial DR (cDRD) (Abrams et al., 2005, Hale et al., 2008). In the study by Christianson et al. (2010), intra-BLA antagonism of the 5-HT_2C_ receptor prevented, while 5-HT_2C_ receptor agonists mimicked, the effects of IS. Inescapable tail shock-induced reduction of 5-HT_1A_ receptor-mediated autoinhibition (Rozeske et al., 2011) may thus explain why only IS/S rats, relative to HC/S rats, displayed increased *tph2* mRNA expression in the dorsomedial DR (cDRD), 4 h following cold swim. The mechanisms through which IS results in decreases in climbing behavior and increases in immobility during forced swimming, 24 h later, remain to be determined.

Cold swim represents both an interoceptive and exteroceptive stressor. Although we did not measure core body temperature in this study, prolonged hypothermia is a component of cold swim stress. For example, while we did not evaluate hypothermic effects of exposure to 15 °C water for 10 min, we have evaluated hypothermic effects of exposure to 19 °C water for 15 min in two separate studies (Kelly et al., 2011, Drugan et al., 2013). Cold swim at 19 °C produced a profound hypothermia, as measured by telemetric recording of core body temperature; core body temperature dropped from approximately 37.6 °C to approximately 27 °C within 10 min (Kelly et al., 2011). Therefore, we conclude that, in the present study, similar responses were involved. In our previous studies, full recovery from hypothermia took approximately 75–100 min, depending on the study. Thus, the prolonged hypothermia, in addition to the exteroceptive/psychological stress of the 10-min cold water swim itself, may have impacted *tph2*, endocrine, and cytokine responses.

It is noteworthy that cold swim stress at 15 °C produces similar behavioral consequences as IS, such as escape deficits, passive stress coping and increased anxiety-like behavior (Christianson and Drugan, 2005). However, unlike IS, by itself, cold swim did not result in elevated *tph2* mRNA expression in the dorsomedial DR (cDRD) 4 h post-stress in our current study. This could be because of the different nature of the stressors (e.g. option of active coping in the water vs. the inescapability of the IS-restraint apparatus), different exposure times (10 min cold swim vs. 100 min IS), the activation of different neuronal networks, or, most likely, a combination of these factors. In previous studies, for example, we have shown that cold swim at 19 °C increases c-Fos expression in serotonergic neurons of the dorsomedial DR (cDRD) (Kelly et al., 2011). Thus, cold swim activates serotonergic neurons acutely, but may be subthreshold for altering *tph2* mRNA expression. Another hypothesis is that the behavioral manifestations of cold swim stress, in the absence of prior IS, depend rather on the noradrenergic system of the locus coeruleus (Detke et al., 1995, Jedema and Grace, 2003, Cryan et al., 2005), while the behavioral effects of IS (although overall similar) depend on dysregulated serotonergic function, such as increased *tph2* expression 4 h post-stress, as well as desensitized 5-HT_1A_ autoreceptors (Rozeske et al., 2011) and increased 5-HT_2C_-mediated serotonergic signaling in the BLA (Christianson et al., 2010) 24 h post-stress. These mechanisms are not mutually exclusive, as locus coeruleus noradrenergic neurons have an excitatory influence on DR serotonergic neurons (Pudovkina et al., 2002).

Consistent with the effect of IS on *tph2* mRNA expression, IS also increased Tph2 protein expression in the DRD (rostral and caudal aspects combined). Based on an estimated time delay of 8 h between peak *tph2* mRNA and peak Tph2 protein expression in the DR (Malek et al., 2004, Malek et al., 2005), we intentionally measured Tph2 protein expression 12 h following the onset of IS, and again 24 h following the onset of IS to assess diurnal variation. Contrary to our expectations, Tph2 protein was only elevated at the 24 h time point, but not at the 12 h time point, in IS rats, compared to HC controls. This may be due to a ceiling effect at the 12 h time point because it falls within the enzyme's natural peak expression during the dark phase. Technical limitations prevented the neuroanatomical microdissection of western blot tissue into rostral and caudal DRD regions, but in the separately dissected DRC, IS abolished the diurnal rhythm of Tph2 protein expression. In comparison, chronic corticosterone treatment also causes anxiety- and depressive-like behavior and has been shown to disrupt the diurnal rhythm of *tph2* mRNA expression in the DRD, DRC and DRV (Donner et al., 2012b).

The finding that *tph2* mRNA expression in the dorsomedial DR (cDRD) was inversely correlated with climbing behavior during forced swimming suggests that serotonergic signaling in the dorsomedial DR (cDRD) may contribute to depressive-like behavioral responses during forced swimming. The fact that this correlation was driven by the HC/S group suggests that otherwise stress-naïve individuals that show inherently passive coping behavior react with higher *tph2* mRNA expression in the dorsomedial DR (cDRD) upon acute stress exposure. Alternatively, individual variability within *tph2* mRNA expression may predict passive coping behavior, with higher *tph2* mRNA expression resulting in increased passive coping behavior. Further studies are required to explore causal relationships.

As expected, rats that were subjected to IS the day before displayed less proactive climbing behavior and more immobility (passive coping behavior). Neurons within the DR are known to be activated by forced swimming (Roche et al., 2003, Commons, 2008, Kelly et al., 2011), but which neurotransmitter systems drive swimming, climbing and immobility behavior is still controversial. Most studies associate an increase in swimming behavior with a stimulated serotonergic system, while increased climbing behavior is attributed to altered dopaminergic or noradrenergic systems (Detke et al., 1995, Cryan et al., 2005, Perona et al., 2008). One hypothesis is that activation of the locus coeruleus selectively activates DRD serotonergic neurons, which are potently activated by α_1_ adrenergic receptors (Baraban and Aghajanian, 1980, Baraban and Aghajanian, 1981, Day et al., 2004).

In another rat model of passive coping during both social defeat and forced swim, peripheral IL-1β expression is elevated (Finnell et al., 2017), while intra-DR expression of IL-1 receptor type 2, a “decoy” receptor and endogenous inhibitor of IL-1, was found to be decreased (Wood et al., 2015), potentially exaggerating a proinflammatory state and depressive-like behavioral responses during forced swimming.

We only measured plasma (not brain) cytokine levels, and did not detect significant effects of IS on IL-1β, but combined exposure to IS and swim stress resulted in increased plasma IL-6 and decreased plasma IL-10 concentrations, indicating an overall proinflammatory milieu. Given that IS leads to a state of behavioral learned helplessness (Maier and Watkins, 2005), these findings match clinical data of depressed patients exhibiting both increased levels of IL-6 while at rest, as well as having greater social stress-induced IL-6 concentrations that normalize after antidepressant therapy (Frommberger et al., 1997, Pace et al., 2006, Fagundes et al., 2013). Plasma IL-6 was also positively correlated with *tph2* mRNA expression in the dorsomedial DR (cDRD), corroborating the hypothesis that proinflammatory cytokines interact with dysregulated neuronal function in psychiatric disease (Raison and Miller, 2013). Interestingly, the correlation was driven by the HC/S group, suggesting that in IS-naïve individuals there is an association between the acute stress-responsiveness of *tph2* mRNA in the dorsomedial DR (cDRD) and that of plasma IL-6. Alternatively, individual variability within *tph2* mRNA expression may predict plasma IL-6 concentrations, with higher *tph2* mRNA expression resulting in increased plasma IL-6 concentrations following S exposure. Further studies are required to explore causal relationships.

Plasma corticosterone was elevated 4 h after cold swim, but not 4 h after IS. Since cold swim procedures constitute a considerable physical challenge on the organism, compared to IS, this discrepancy is not surprising. Glucocorticoid signaling after cold swim is probably needed for a longer period of time to mobilize energy and recover from hypothermia, given that the animals are recovering from a 10-min long suspension in 15 °C water (Steffen and Musacchia, 1985). Work by Kelly et al. (2011), furthermore, indicates that mild hyperthermic responses occur in rats 90–130 min (or longer) following swimming in water temperatures below 19 °C, corroborating the physical challenge of recovering from cold swimming. It is unclear why IS resulted in a delayed increase in plasma corticosterone 28 h after the onset of IS (in the IS/HC group). While the possibility of a cued corticosterone response in the IS/HC group (e.g., a response to the experimenter entering the housing room on Day 2 to retrieve the swim group animals) exists, it is known that IS induces a long-lasting increase in basal corticosterone that can last for up to 48–96 h post-stress (Fleshner et al., 1995). The measured elevation at 28 h may simply reflect this increase, and indicate the beginning of a chronic dysregulation in a hypothalamic control center (O'Connor et al., 2004). It is unlikely that the lack of further elevation of plasma corticosterone in the IS/S group is due to a ceiling effect because a) stress-induced corticosterone levels easily reach 500 ng/ml whereas the IS/S group didn't exceed 100 ng/ml, b) 4 h post-swim the corticosterone stress response was most likely beyond its descending limb (Rittenhouse et al., 2002), and c) IS is known to lead to a sensitization of HPA axis responses to a subsequent challenge (Johnson et al., 2003, O'Connor et al., 2004).

A limitation of the current work is that, for logistical reasons, corticosterone and cytokine data endpoints were examined at time points optimized for *tph2* mRNA responses to stress (i.e., 4 h after the onset of IS or cold swim). The key experiment was an examination of the ability of IS to alter serotonergic, endocrine and cytokine responses to a heterotypic stressor, cold swim. These two stressors very likely produce changes in corticosterone and cytokine levels with differing magnitudes and time-courses. While collecting *tph2* mRNA, corticosterone, and cytokine data at the same time point allows comparisons among these parameters in the context of stress, it doesn't address the complete time window of the endocrine or cytokine responses to the stressors. To answer the question of whether the corticosterone or cytokine response to swim is changed by prior IS-exposure, corticosterone and cytokine levels would need to be determined in the four experimental groups across the full time-course of the experiment (e.g. maximum effect, area-under-the-curve measurements, etc.). For example, corticosterone levels were analyzed 4 h following the onset of the 10-min cold swim procedure, a time point that may well be on the descending limb of the endocrine response (Rittenhouse et al., 2002) and therefore not a good overall representation of that response. Despite these limitations, increased IL-6 concentrations, as observed in the IS/S group, are consistent with elevation of IL-6 in humans exposed to trauma, based on a trans-diagnostic meta-analysis (Tursich et al., 2014), and with rodent studies indicating that individual variability in IL-6 mediates stress vulnerability (Hodes et al., 2014).

## 5 Conclusion

Together, these data are consistent with the hypothesis that inescapable or uncontrollable stressors, such as IS, lead to a proinflammatory cytokine milieu and increase *tph2* mRNA expression in serotonergic neurons within the DRD, specifically the dorsomedial DR (cDRD). Inescapable stress causes a region-specific increase of *tph2* mRNA and Tph2 protein, and is likely to mediate diverse behavioral consequences, including anxiety-related and depressive-like behavior. These findings are also of clinical relevance because the brains of depressed suicide victims display elevated *TPH2* mRNA expression (Bach-Mizrachi et al., 2006, Bach-Mizrachi et al., 2008) and increased TPH2 immunoreactivity (Boldrini et al., 2005, Bonkale et al., 2006), in part within comparable dorsomedial regions of the human DR (Bach-Mizrachi et al., 2006, Bach-Mizrachi et al., 2008, Bonkale et al., 2006, Bach-Mizrachi et al., 2008).

There is considerable debate over whether the 5-HT system is hypoactive or hyperactive in depression. Much of the belief comes from the clinical effects of 5-HT-promoting therapies in the form of serotonin reuptake inhibitors (SSRIs) which, at least acutely, enhance serotonergic signaling at the level of the synapse. However, when brain 5-HT turnover was directly measured in depressed patients by Esler and colleagues (Barton et al., 2008), brain 5-HT turnover was elevated in unmedicated depressed patients, and this effect was larger in patients carrying the *s* allele, relative to those carrying the *l* allele of the polymorphic region of the serotonin transporter promoter (for review, see Andrews et al. (2015)). Furthermore, SSRI treatment reduced brain 5-HT turnover to normal levels. We suggest that 5-HT turnover in some serotonergic systems (for example, serotonergic neurons within the dorsomedial DR (cDRD) projecting to the BLA and driving increased anxiety-like behavioral responses through actions on 5-HT_2C_ receptors (Christianson et al., 2010)), are hyperactive, while those projecting to the medial prefrontal cortex and thought to be involved in stress recovery or resolution of stress responses are hypoactive (Forster et al., 2006, Forster et al., 2008).

## Declaration of interest

The authors report no conflict of interest. This work was supported by the National Institutes of Health [grant numbers R01 MH086539 (CAL) and R01 MH108523 (SFM)].

## Appendix A Supplementary data

Data profile Data profile
