# Supplementary material for: Two models of inescapable stress increase tph2 mRNA expression in the anxiety-related dorsomedial part of the dorsal raphe nucleus
Source: Neurobiol Stress. 2018 Jan 17;8:68–81. doi: 10.1016/j.ynstr.2018.01.003 (PMC5842308; doi:10.1016/j.ynstr.2018.01.003)
Supplement: Supplemental Materials [file mmc2.pdf]

Supplemental Materials

Manuscript Title: Two models of inescapable stress increase *tph2* mRNA expression in the anxiety-related dorsomedial part of the dorsal raphe nucleus

Authors: Nina C. Donner, Kenneth H. Kubala, James E. Hassell Jr., Margaret W. Lieb, Kadi T. Nguyen, Jared D. Heinze, Robert C. Drugan, Steven F. Maier, Christopher A. Lowry

1. Supplemental Methods

1.1. Choice of stressor type and time points for brain collection

The choice of IS as a stressor is based on previous work showing that the behavioral consequences of IS are dependent on functional desensitization of 5-HT$_{1A}$ inhibitory autoreceptors within the dorsal part of the dorsal raphe nucleus (Rozeske *et al.* 2011), and sensitization of serotonergic systems projecting to the basolateral amygdala 24 h later (Christianson *et al.* 2010). These effects have been documented in previous studies using *in vivo* microdialysis in the dorsal raphe nucleus and the amygdala, and validated using pharmacological approaches to prevent the behavioral effects of IS using 5-HT receptor agonists and antagonists (Maier and Watkins 1998, Maier and Watkins 2005). Furthermore, the afferent systems controlling the serotonergic neurons during IS have been documented, and include the bed nucleus of the stria terminalis (Hammack *et al.* 2004) and lateral habenula (Dolzani *et al.* 2016). This extensive knowledge of the biological mechanisms underlying the behavioral consequences of IS was the basis of our choice of IS as a stressor. There is no other stressor for which this depth of knowledge is available.

The time points for measurement of *tph2* mRNA expression in *Experiments 1* and *3* (4 h following stress onset) were chosen because other paradigms lead to increases in *tph2* mRNA at this time point (Gardner et al., 2009). The first time point for measurement of Tph2 protein (12 h following the onset of IS) in *Experiment 2* was selected because Malek et al. demonstrated in a series of studies that peak expression of Tph2 protein occurs approximately 8 h following peak *tph2* mRNA expression during normal diurnal variation of expression in rats (Malek *et al.* 2004, Malek *et al.* 2005, Malek *et al.* 2007), and we also previously observed increases in rat Tph2 protein expression and rat Tph2 activity 8 h following the diurnal increase of *tph2* mRNA expression (Donner *et al.* 2016). The second time point for measurement of Tph2 protein (24 h following the onset of IS) was included to assess how long a potential effect of IS on Tph2 protein expression would last, to detect potential alterations of the natural diurnal variation of the enzyme, and to determine Tph2 protein expression at the time of behavioral testing during cold swim exposure.

1.2. Inescapable tail shock procedure

Inescapable tail shock (IS) consisted of intermittent inescapable tail shocks administered in a sound-proof room within clear acrylic restraint tubes that measured 17 cm in length and 7 cm in diameter. Each restraint tube contained a small platform extending from the rear to which the rat's tail was fixed with cloth tape and electrodes. The IS procedure consisted of 100 trials of 5 s shocks on a variable interval schedule (within a 50 to 60 second time window per trial) at an intensity of 1.0 mA for the first 10 shocks, 1.3 mA for the second 10 shocks and 1.6 mA for all subsequent trials as previously reported (Christianson *et al.* 2008, Christianson *et al.* 2011). The IS session lasted for about 100 min, and rats were returned to their home cages immediately following the tail-shock procedure. Stress-naïve control rats remained undisturbed in their home cages (HC).

1.3. Cold swim stress and behavioral analysis

In *Experiment 3,* rats were exposed to either HC or IS conditions, then, 24 h later, exposed to either HC control conditions or a 10-min session of cold swim stress (S) at 15 °C water temperature in a Pyrex® glass cylinder measuring 45.7 cm in height and 30.5 cm in diameter (Cat No. 36360-201, VWR, West Chester, PA, USA) with a water depth of 30 cm, ensuring that the rats were unable to balance on the bottom of the cylinder using their tails. Fresh water was used for each rat. After the forced swim procedure, rats were rubbed dry thoroughly with a towel, and returned to their home cages and colony housing room for recovery. To avoid the potential confound of activation of warm-sensitive serotonergic systems (Hale *et al.* 2011), rats were not placed on heating pads during recovery. The rats' behavior during the 10-min test was recorded using a digital video camera that was placed in front of the glass cylinder, allowing for a sideways view, slightly above the waterline. Analysis of forced swim test data was conducted as described

previously (Cryan *et al.* 2005, Kelly *et al.* 2011, Donner *et al.* 2012, Drugan *et al.* 2013, Hale *et al.* 2017). Using the behavioral scoring software The Observer XT, version 5.0 (Noldus Information Technology, Wageningen, The Netherlands), we manually scored the time spent climbing (proactive coping behavior involving high-pace front leg paddling that breaks the water surface and strong hind leg strokes), the time spent swimming (proactive coping behavior involving slow-paced front- and hind-leg movements, no breaking of the water surface), the time spent immobile (reactive, despair-like behavior involving minimal hind leg movements required only to stay afloat and keep the nose above water, and stiff body posture), and counted the number of dives (interpretable as proactive, explorative escape-seeking behavior). The observer was blinded to the treatment groups during scoring. Start- and stop-keys on the computer keyboard representing "climbing", "swimming" and "immobility" were used to assess the time spent with each behavior during the cold swim procedure. Dives were entered as single events, using a different key. We also conducted an inter-rater reliability analysis based on behavioral scoring of the entire data set by two separate investigators (NCD and JDH), both blinded to the treatment groups. The analysis was conducted for individual behaviors, using Pearson correlation with all observations. Analysis revealed high inter-rater reliability for all behaviors in *Experiment 3*: (1) climbing ($r = 0.978$; $p < 0.001$); (2) swimming ($r = 0.997$; $p < 0.001$); (3) immobility ($r = 0.994$; $p < 0.001$). Based on the high inter-rater reliability of behavioral scoring, only behavioral scores conducted by a single investigator (NCD) are presented.

1.4. Plasma corticosterone concentrations

Total plasma corticosterone concentrations were analyzed with an enzyme-linked immunoassay kit from Assay Designs (Cat. No. 900-097, Ann Arbor, MI, USA). According to the manufacturer, this assay has a sensitivity of 0.027 ng/ml, an intra-assay coefficient of variation (CV) of 6 to 8%, and an inter-assay CV of 8 to 13%. Empirically, we determined an intra-assay coefficient of variation of 8.28% and an inter-assay CV of 11.03%. Prior to being analyzed in duplicate, samples were diluted 1:40 in assay buffer containing 2.5% steroid displacement reagent (provided by the manufacturer). Samples from *Experiment 1* and *3* were run on two different 96-well plates.

1.5. Plasma cytokine concentrations (IL-1β, IL-6 and IL-10)

Plasma concentrations of the pleiotropic cytokine interleukin (IL)-6 were determined in undiluted duplicate samples with an enzyme-linked immunoassay from Invitrogen (Cat. No. KRD0061C, Carlsbad, CA, USA). This IL-6 assay has a range of 5.0 to 1500 pg/ml, an empirically determined intra-assay CV of 7.96% (manufacturer-reported value: 3-6%) and an inter-assay CV of 7.06% (manufacturer-reported value: 5-9%). Samples from *Experiment 1* and *3* were run on two different 96-well plates. Plasma concentrations of the proinflammatory cytokine IL-1β were measured as singlets (due to restricted availability of plasma volume) in undiluted plasma on one 96-well plate, using an enzyme-linked immunoassay (Cat. No. ELR-IL1beta-001, RayBiotech, Inc., Norcross, GA, USA) with a sensitivity of 80 pg/ml, an empirically determined intra-assay CV of 8.06% (average of % CVs of standards that were measured in duplicate), and a manufacturer-reported inter-assay CV of less than 12%. Concentrations of the anti-inflammatory cytokine IL-10 were also measured as singlets (due to restricted availability of plasma volume) in undiluted plasma on one 96-well plate, using an enzyme-linked immunoassay (Cat. No. ELR-IL10-001, RayBiotech, Inc., Norcross, GA, USA) with a sensitivity of 10 pg/ml, an empirically determined intra-assay CV of 4.15% (average of % CVs of standards that were measured in duplicate) and a manufacturer-reported inter-assay CV of less than 12%. In case of the IL-1β and IL-10 assays, many sample values were lower than the manufacturer-reported sensitivity of the respective assay, but still fell on the standard-based four-parameter logistic curve, and were thus used for statistical analysis. Values below the curve were set to zero, but also included in the analysis.

1.6. Details of in situ *hybridization histochemistry for* tph2 *mRNA and semi-quantitative analysis of* tph2 *mRNA expression*

Brain sections were thawed at room temperature, fixed with 4% paraformaldehyde in 0.05 M phosphate-buffered saline (PBS) for 10 min, and rinsed three times in 2X standard saline citrate buffer (SSC) for 10 min each. Hybridization solution (90 µl/slide, 0.4 µg/ml radiolabeled probe, 25 mM Tris, pH 7.4, 40% deionized formamide, 500 µg/ml single-stranded salmon sperm DNA, 250 µg/ml transfer RNA, 1X Denhardt's solution, 4 mM ethylenediaminetetraacetic acid (EDTA), 5 mM sodium chloride, 10% dextran sulfate) was dispensed on each slide, while avoiding air bubbles, and resulting in about 1 x 10^6 cpm of radiolabeled probe per slide. Rectangular parafilm pieces were used as coverslips, and slides were incubated overnight in a humidified chamber at 55 °C. The next day, coverslips were removed before all

slides were washed once in 2X SSC and three times in 1X SSC, for 10 min each. To remove unbound riboprobe and nonspecifically bound riboprobe fragments, slides were then incubated in ribonuclease-A solution (0.05 M Tris-Cl, 0.025 M EDTA, 0.5 M NaCl and 20 µg/ml RNase A) for 1 h at 37 °C. After this, slides were washed in 1X SSC containing 1 ml/L beta-mercaptoethanol for 30 min at room temperature, and then in 1X SSC containing 1 ml/L beta-mercaptoethanol for 30 min at 60 °C. Lastly, slides were desalted in 0.5X and 0.1X SSC containing 1 ml/L beta-mercaptoethanol for 10 min each, and then gradually dehydrated in 50%, 70%, 90% and 100% ethanol containing 0.3 M ammonium acetate. After air-drying for 20 min, all slides were apposed to a Kodak BioMax autoradiography film (PerkinElmer, Waltham, MA) for 7 days.

1.7. Details of Tph2 western blot

Total protein content was determined in 5 µl of each sample, using a commercially available, colorimetric protein assay (Micro BCA Protein Assay Kit, Cat. No. 1856210, Thermo Scientific), while 30 µg protein per sample were mixed with 2X clear loading buffer (pH 6.5, 0.4 M Tris, 20% glycerol, 4% sodium dodecyl sulfate (SDS), 2% mercaptoethanol in ddH_2O) and 7% loading dye, boiled for 3 min, quenched on ice, and then separated in a gel electrophoresis chamber (Cat. No. 4561045, BioRad, Hercules, CA, USA) for 105 min at 90 V, using a precast 12% SDS-polyacrylamide gel (Cat. No. 456-1043, BioRad) and 1X of a Tris/glycine running buffer (Cat. No.161-0734EDU, BioRad). Two columns per gel were loaded with a 10 to 250 kDa protein ladder (Cat. No. 161-0373, BioRad) for later band identification (molecular weight of rat Tph2 protein: 56 kDa (Manjarrez-Gutierrez *et al.* 2012)). Protein bands were blotted from the gel onto a 0.2 µm Immun-Blot® PVDF membrane (Cat. No. 162-0176, BioRad) for 2 h at 60 mA at 4 °C, after soaking the membrane briefly in methanol and the blotting paper (Cat. No. 170-3955, BioRad) in transfer buffer (0.58% Trizma base, 0.29% glycine, 0.04% SDS, 20% methanol). The PVDF membrane was washed in ddH_2O, incubated with SuperSignal® Western Blot Enhancer antigen pretreatment solution (Cat. No. 46640, ThermoScientific, Rockford, IL, USA) for 10 min, and rinsed in ddH_2O. After 20 min of blocking in 1X Tris-buffered saline/Tween (TBST; 0.12% Trizma base, 0.58% NaCl, 0.1% Tween 20) with 5% dry milk powder, the membrane was incubated with SuperSignal® Western Blot Enhancer primary antibody solution (Cat. No. 46640, ThermoScientific) including the antibody against Tph2 (1:200 rabbit anti-rat, Cat. No. ABN60, Millipore) at 4 °C overnight, slowly shaking. Membranes were washed in TBST buffer, and incubated with horseradish-peroxidase-conjugated secondary antibody for 2h at RT (1:500, Cat. No. AP182P, Millipore) in 5% dry milk TBST. After a final TBST wash, the membrane was then exposed to a chemoluminescent kit (ECL^{TM}, Cat. No. RPN2106, GE Healthcare, Pittsburgh, PA, USA) for 1 min at RT. The blot was visualized using the "ChemiDoc-It" image-capturing system (Model No. 102606-011, UVP Bioimaging Systems, Upland, CA, USA), the software VisionWorks®LS (version 5.5, UVP Bioimaging Systems), and 20-40 min capture time. No Tph2 protein expression was detected in the pineal gland (negative control microdissection), verifying primary antibody specificity for Tph2 relative to Tph1.

Detection of β-actin (molecular weight: 42 kDa) as a loading control occurred after rinsing the membrane in TBST. A goat anti-mouse/rat/human primary antibody (1:3500 dilution at 4 °C overnight, Cat. No. MAB1501R, Millipore) and a horseradish-peroxidase-conjugated secondary antibody (1:500 dilution for 2 h at RT, Cat. No. A3682, Sigma-Aldrich) were used in the same procedure as described above. Blotted bands were visualized using chemoluminescence followed by 5 min image acquisition time.

2. Supplemental Results

2.1. Detailed statistics and graphs for tph2 *mRNA and Tph2 protein expression in Experiment 1, Experiment 2 and Experiment 3*

Supplemental Tables 1, 2 and 3, as well as Supplemental Figures 1, 2 and 3, display detailed overall and subdivision-specific statistical results of individual LMM analyses (or ANOVAs for subdivision-specific Tph2 protein data sets without repeated measures), including bar graphs summarizing results for each anatomical subdivision of the DR, and graphs illustrating *tph2* mRNA expression throughout the rostrocaudal extent of the DR and each subdivision of the DR.

2.2. Correlation of tph2 *mRNA expression in the DRD with climbing behavior and plasma IL-6*

Correlation analysis revealed an inverse relationship between *tph2* mRNA expression in the dorsomedial DR (cDRD) and climbing behavior during the cold swim procedure (Supplemental Fig. 4a; $p < 0.001$, $r^2 = 0.562$, $N = 16$). High *tph2* mRNA expression within the dorsomedial DR (cDRD) was associated

with decreased proactive coping behavior (in the form of decreased percent time spent climbing; Supplemental Fig. 4a). Within-group regression analysis furthermore revealed that this correlation was driven by the "naïve" HC/S group, not the "stress-experienced" IS/S group (Supplemental Fig. 4d, $p < 0.05$, $r^2 = 0.562$, $n = 8$). Neither immobility nor swimming behavior was correlated with *tph2* mRNA expression in any of the DR subdivisions. Regression analysis of *tph2* mRNA expression in *Experiment 3* and plasma cytokine concentrations revealed a positive correlation of *tph2* mRNA in the entire DRD and plasma IL-6 (Supplemental Fig. 4b; $p < 0.05$, $r^2 = 0.130$, $N = 32$), and, more specifically, of *tph2* mRNA expression in the dorsomedial DR (cDRD) and plasma IL-6 (Supplemental Fig. 4C; $p < 0.05$, $r^2 = 0.131$, $N = 32$). In both cases, these correlation effects were, again, driven by the HC/S group (Supplemental Fig. 4e; $p = 0.065$, $r^2 = 0.458$, $n = 8$; and Supplemental Fig. 4f; $p < 0.05$, $r^2 = 0.576$, $n = 8$). For *Experiment 1*, regression analysis of *tph2* mRNA expression in the DRI and IL-10 approached significance ($r^2 = 0.260$, $p = 0.062$, $N = 16$), with rats that expressed more *tph2* mRNA having, in tendency, a lower concentration of plasma IL-10. No other correlations between subdivision-specific *tph2* mRNA expression and plasma cytokines or plasma corticosterone were found.

3. Supplemental Tables and Figures

Supplemental Table 1. Linear mixed model analysis for *Experiment 1* (*tph2* mRNA)

Model	Source	Test statistic	*p*-value
**Overall analysis**			
**Entire DR (First-Order Ante-Dependence Covariance Structure)**			
	Inescapable shock	$F_{(1, 76.7)} = 5.62$	0.020*
	Rostrocaudal level	$F_{(12,38.4)} = 13.03$	0.001***
	DR subdivision	$F_{(6,64.1)} = 21.72$	0.001***
	Inescapable shock * Rostrocaudal level	$F_{(12,38.4)} = 0.59$	0.840
	Inescapable shock * DR subdivision	$F_{(6,64.1)} = 0.10$	0.997
	Rostrocaudal level (DR subdivision)	$F_{(18,35.5)} = 12.43$	0.001***
	Inescapable shock * Rostrocaudal level (DR subdivision)	$F_{(18,35.5)} = 0.62$	0.856
**Analysis of each DR subdivision**			
**rDRD (Diagonal Covariance Structure)**			
	Inescapable shock	$F_{(1,18.1)} = 0.99$	0.331
	Rostrocaudal level	$F_{(4,13.6)} = 2.76$	0.071
	Inescapable shock * Rostrocaudal level	$F_{(4,13.6)} = 0.21$	0.929
**cDRD (Diagonal Covariance Structure)**			
	Inescapable shock	$F_{(1,30.9)} = 9.94$	0.004**
	Rostrocaudal level	$F_{(4,20.9)} = 5.57$	0.003**
	Inescapable shock * Rostrocaudal level	$F_{(4,20.9)} = 0.52$	0.720
**rDRV (Diagonal Covariance Structure)**			
	Inescapable shock	$F_{(1,25.4)} = 0.38$	0.544
	Rostrocaudal level	$F_{(4,9.93)} = 15.17$	0.001***
	Inescapable shock * Rostrocaudal level	$F_{(4,9.93)} = 0.21$	0.930
**cDRV (Diagonal Covariance Structure)**			
	Inescapable shock	$F_{(1,34.4)} = 3.07$	0.089
	Rostrocaudal level	$F_{(6,20.2)} = 15.63$	0.001***
	Inescapable shock * Rostrocaudal level	$F_{(6,20.2)} = 0.17$	0.949
**DRVL / VLPAG (Unstructured Covariance Structure)**			
	Inescapable shock	$F_{(1,13.8)} = 2.18$	0.162
	Rostrocaudal level	$F_{(8,14.3)} = 74.57$	0.001***
	Inescapable shock * Rostrocaudal level	$F_{(8,14.3)} = 1.02$	0.462
**DRI (Unstructured Covariance Structure)**			
	Inescapable shock	$F_{(1,12.5)} = 2.21$	0.162
	Rostrocaudal level	$F_{(2,11.2)} = 18.26$	0.001***
	Inescapable shock * Rostrocaudal level	$F_{(2,11.2)} = 2.92$	0.095
**DRC (Unstructured Covariance Structure)**			
	Inescapable shock	$F_{(1,13.4)} = 0.20$	0.664
	Rostrocaudal level	$F_{(2,11.8)} = 93.67$	0.001***
	Inescapable shock * Rostrocaudal level	$F_{(2,11.8)} = 5.32$	0.022*

Abbreviations: cDRD, dorsomedial DR (caudal aspect of the dorsal raphe nucleus, dorsal part); cDRV, caudal aspect of the dorsal raphe nucleus, ventral part; DRC, dorsal raphe nucleus, caudal part; DRI, dorsal raphe nucleus, interfascicular part; DRVL, dorsal raphe nucleus, ventrolateral part; rDRD, rostral aspect of the dorsal raphe nucleus, dorsal part; rDRV, rostral aspect of the dorsal raphe nucleus, ventral part; VLPAG, ventrolateral periaqueductal gray.

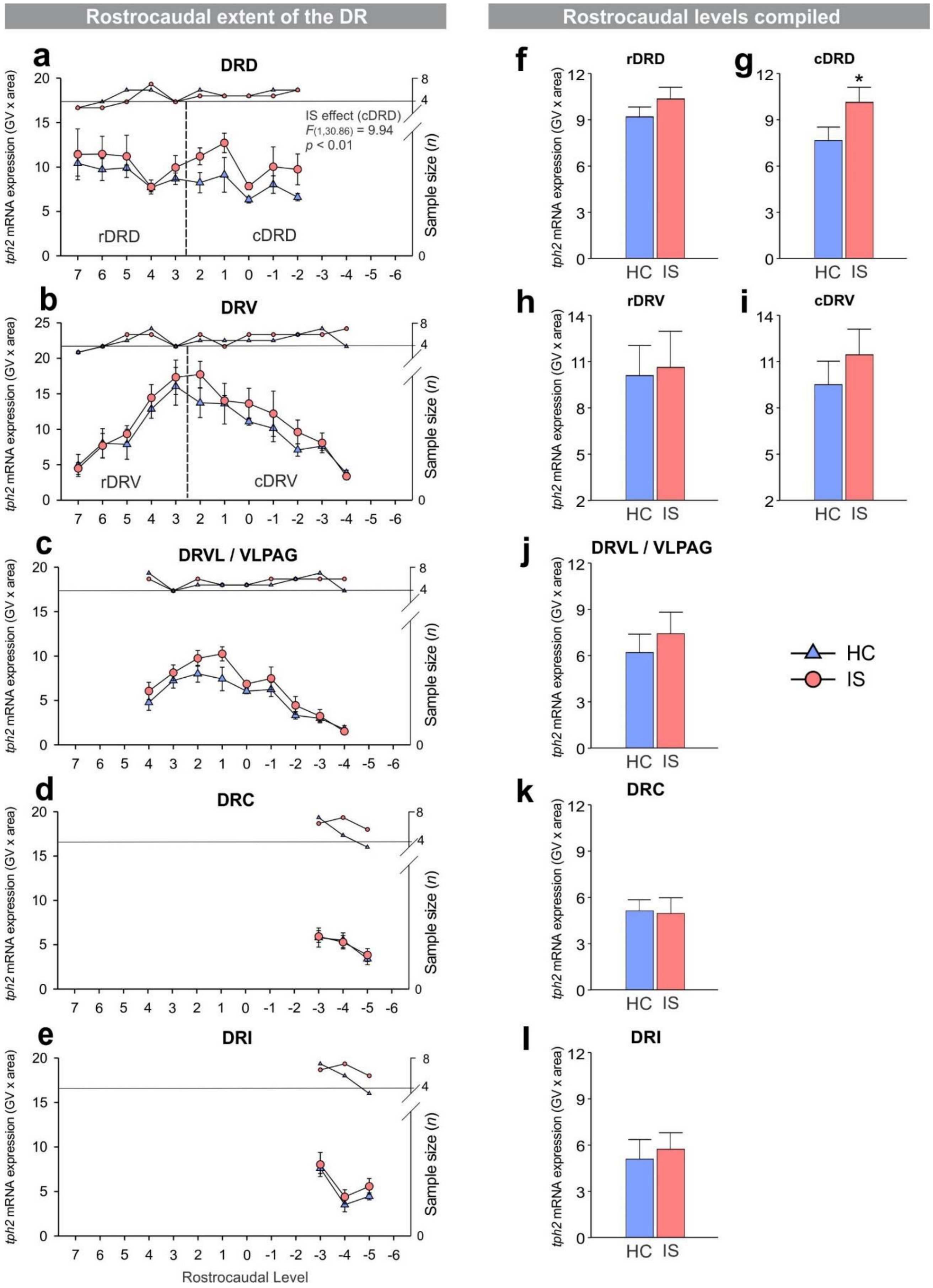

Supplemental Figure 1. *tph2* mRNA expression 4 h following exposure to inescapable tail shock (IS) or home cage control conditions (HC). Graphs on the left side (**a-e**) represent the mean ± SEM of *tph2* mRNA expression throughout the rostrocaudal extent of each DR subdivision, graphs on the right side (**f-l**) represent the overall *tph2* mRNA expression + SEM per subdivision (rostrocaudal levels averaged) measured 4 h after the onset of IS or after HC control conditions, respectively, in *Experiment 1*. All rats were euthanized 4 h after the onset of IS (IS, n = 8), including unstressed control rats (HC, n = 8). Graphs illustrate *tph2* mRNA expression in the (**a**) dorsal raphe nucleus, dorsal part (DRD), (**f**) rostral aspect of the DRD (rDRD), (**g**) dorsomedial DR (caudal aspect of the DRD (cDRD)), (**b**) dorsal raphe nucleus, ventral part (DRV), (**h**) rostral aspect of the DRV (rDRV), (**l**) caudal aspect of the DRV (cDRV), (**c**) dorsal raphe nucleus, ventrolateral part (DRVL) / ventrolateral periaqueductal gray (VLPAG), (**d**) dorsal raphe nucleus, caudal part (DRC), and (**e**) dorsal raphe nucleus, interfascicular part (DRI). *Post hoc* comparisons were made using Student's *t*-tests. *Post hoc* testing was not conducted at a specific rostrocaudal level (7 through -6) when 1 or more groups contained less than half of the full sample size, indicated by the right y-axis. No significant treatment effects were found at individual rostrocaudal levels. Rostrocaudal levels 7 = -7.580 mm, 6 = -7.664 mm, 5 = -7.748 mm, 4 = -7.832 mm, 3 = -7.916 mm, 2 = -8.000 mm, 1 = -8.084 mm, 0 = -8.168 mm, -1 = -8.252 mm, -2 = -8.336 mm, -3 = -8.420 mm, -4 = -8.504 mm, -5 = -8.588 mm, -6 = -8.672 mm from bregma.

Supplemental Table 2. Statistical analysis for *Experiment 2* (Tph2 protein)

Model	Source	Test statistic	*p*-value
**Overall analysis**			
**Entire DR (Linear Mixed Model, Unstructured Covariance)**			
	Inescapable shock	$F_{(1, 28.7)} = 6.00$	0.021*
	Time point (12 h vs 24 h)	$F_{(1, 28.7)} = 9.56$	0.004**
	DR subdivision	$F_{(4, 27.2)} = 21.87$	0.001***
	Inescapable shock * Time point (12 h vs. 24 h)	$F_{(1, 28.7)} = 0.06$	0.813
	Inescapable shock * DR subdivision	$F_{(4, 27.2)} = 2.86$	0.049*
	Time point (12 h vs. 24 h) * DR subdivision	$F_{(4, 27.2)} = 2.42$	0.073
	Inescapable shock * Time point * DR subdivision	$F_{(4, 27.2)} = 0.27$	0.898
**Analysis of each DR subdivision (samples of rostrocaudal levels pooled)**			
**DRD (2 x 2 ANOVA)**			
	Inescapable shock	$F_{(1, 28)} = 4.61$	0.041*
	Time point (12 h vs. 24 h)	$F_{(1, 28)} = 7.58$	0.010**
	Inescapable shock * Time point (12 h vs. 24 h)	$F_{(1, 28)} = 0.001$	0.979
**DRV (2 x 2 ANOVA)**			
	Inescapable shock	$F_{(1, 26)} = 0.95$	0.339
	Time point (12 h vs. 24 h)	$F_{(1, 26)} = 1.18$	0.287
	Inescapable shock * Time point (12 h vs. 24 h)	$F_{(1, 26)} = 0.21$	0.648
**DRVL / VLPAG (2 x 2 ANOVA)**			
	Inescapable shock	$F_{(1, 28)} = 1.74$	0.198
	Time point (12 h vs. 24 h)	$F_{(1, 28)} = 0.03$	0.867
	Inescapable shock * Time point (12 h vs. 24 h)	$F_{(1, 28)} = 0.29$	0.597
**DRI (2 x 2 ANOVA)**			
	Inescapable shock	$F_{(1, 28)} = 1.15$	0.293
	Time point (12 h vs. 24 h)	$F_{(1, 28)} = 1.66$	0.208
	Inescapable shock * Time point (12 h vs. 24 h)	$F_{(1, 28)} = 0.002$	0.963
**DRC (2 x 2 ANOVA)**			
	Inescapable shock	$F_{(1, 25)} = 0.02$	0.886
	Time point (12 h vs. 24 h)	$F_{(1, 25)} = 4.02$	0.056
	Inescapable shock * Time point (12 h vs. 24 h)	$F_{(1, 25)} = 0.56$	0.461

Abbreviations: DRC, dorsal raphe nucleus, caudal part; DRD, dorsal raphe nucleus, dorsal part; DRI, dorsal raphe nucleus, interfascicular part; DRV, dorsal raphe nucleus, ventral part; DRVL, dorsal raphe nucleus, ventrolateral part; VLPAG, ventrolateral periaqueductal gray.

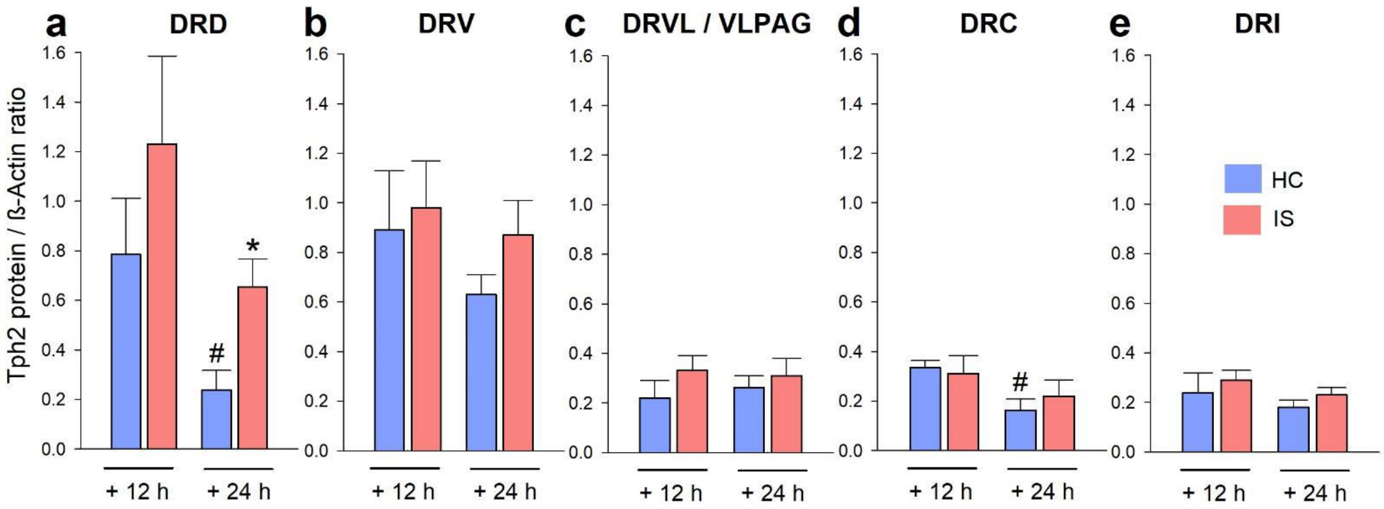

Supplemental Figure 2. Tph2 protein expression 12 h and 24 h following inescapable tail shock (IS) in all DR subdivisions. Displayed is the expression of tryptophan hydroxylase 2 (Tph2) protein in all anatomical subdivisions of the DR (rostrocaudal samples had to be pooled) either 12 h or 24 h after the onset of IS, compared to home cage (HC) control conditions (**a**-**e**; HC/12 h, *n* = 8; IS/12 h, *n* = 8; HC/24 h, *n* = 8; IS/24 h, *n* = 8). Beta-actin (β-Actin) was used as the loading control during the western blot assay. Abbreviations: (**a**) DRD, dorsal raphe nucleus, dorsal part; (**b**) DRV, dorsal raphe nucleus, ventral part; (**c**) DRVL / VLPAG, dorsal raphe nucleus, ventrolateral part / ventrolateral periaqueductal gray; (**d**) DRC, dorsal raphe nucleus, caudal part; (**e**) DRI, dorsal raphe nucleus, interfascicular part. Data represent means + SEM. *$p < 0.05$ vs. the HC control group of the same time point (IS effect); $^{\#}p < 0.05$ vs. the 12 h time point of the same treatment group (diurnal rhythm effect). Pairwise *post hoc* comparisons were performed using the Student´s *t*-test for independent samples.

Supplemental Table 3. Linear mixed model analysis for *Experiment 3* (*tph2* mRNA)

Model	Effect Type	Test statistic	*p*-value
**Overall analysis**			
**Entire DR (First-Order Ante-Dependence Covariance Structure)**			
	Inescapable shock	$F_{(1, 221.9)}$ = 14.61	0.001***
	Cold swim	$F_{(1, 221.9)}$ = 9.54	0.002**
	Rostrocaudal level	$F_{(10,103,7)}$ = 25.33	0.001***
	DR subdivision	$F_{(6, 215.9)}$ = 53.90	0.001***
	Inescapable shock * Cold swim	$F_{(1,251.0)}$ = 0.30	0.586
	Inescapable shock * Rostrocaudal level	$F_{(10,103.7)}$ = 2.20	0.023*
	Inescapable shock * DR subdivision	$F_{(6, 215.9)}$ = 1.15	0.336
	Cold swim * Rostrocaudal level	$F_{(10, 103.7)}$ = 1.10	0.367
	Cold swim * DR subdivision	$F_{(6, 215.9)}$ = 0.58	0.746
	Inescapable shock * Rostrocaudal level (DR subdivision)	$F_{(22, 243.8)}$ = 0.70	0.836
	Cold swim * Rostrocaudal level (DR subdivision)	$F_{(22, 243.8)}$ = 1.25	0.207
	Inescapable shock * Cold swim * Rostrocaudal level (DR subdivision)	$F_{(38,161.7)}$ = 0.73	0.877
**Analysis of each DR subdivision**			
**rDRD (First-Order Ante-Dependence Covariance Structure)**			
	Inescapable shock	$F_{(1,44.2)}$ = 0.69	0.410
	Cold swim	$F_{(1,44.2)}$ = 1.35	0.251
	Rostrocaudal level	$F_{(4,36.5)}$ = 5.35	0.002**
	Inescapable shock * Cold swim	$F_{(1,44.2)}$ = 0.24	0.630
	Inescapable shock * Rostrocaudal level	$F_{(4,36.5)}$ = 0.28	0.890
	Cold swim * Rostrocaudal level	$F_{(4,36.5)}$ = 0.88	0.485
	Inescapable shock * Cold swim * Rostrocaudal Level	$F_{(4,36.5)}$ = 0.99	0.424

**cDRD (First-Order Ante-Dependence Covariance Structure)**		
Inescapable shock	$F_{(1,29.1)} = 10.20$	0.003**
Cold swim	$F_{(1,29.1)} = 4.50$	0.043*
Rostrocaudal level	$F_{(4,24.3)} = 10.99$	0.001***
Inescapable shock * Cold swim	$F_{(1,29.1)} = 0.22$	0.646
Inescapable shock * Rostrocaudal level	$F_{(4,24.3)} = 0.94$	0.460
Cold swim * Rostrocaudal level	$F_{(4,24.3)} = 0.55$	0.699
Inescapable shock * Cold swim * Rostrocaudal Level	$F_{(4,24.3)} = 0.40$	0.806
**rDRV (First-Order Ante-Dependence Covariance Structure)**		
Inescapable shock	$F_{(1,33.2)} = 0.37$	0.545
Cold swim	$F_{(1,33.2)} = 2.10$	0.157
Rostrocaudal level	$F_{(4,39.5)} = 36.42$	0.001***
Inescapable shock * Cold swim	$F_{(1,33.2)} = 0.20$	0.659
Inescapable shock * Rostrocaudal level	$F_{(4,39.5)} = 0.32$	0.863
Cold swim * Rostrocaudal level	$F_{(4,39.5)} = 1.24$	0.311
Inescapable Shock * Cold swim * Rostrocaudal Level	$F_{(4,39.5)} = 0.83$	0.513
**cDRV (First-Order Ante-Dependence Covariance Structure)**		
Inescapable shock	$F_{(1,32.6)} = 4.41$	0.044*
Cold swim	$F_{(1,32.6)} = 0.91$	0.347
Rostrocaudal level	$F_{(6,39.2)} = 9.70$	0.001***
Inescapable shock * Cold swim	$F_{(1,32.6)} = 0.11$	0.745
Inescapable shock * Rostrocaudal level	$F_{(6,39.2)} = 0.80$	0.578
Cold swim * Rostrocaudal level	$F_{(6,39.2)} = 0.53$	0.780
Inescapable shock * Cold swim * Rostrocaudal Level	$F_{(6,39.2)} = 0.40$	0.872
**DRVL / VLPAG (Unstructured Covariance Structure)**		
Inescapable Shock	$F_{(1,26.1)} = 3.61$	0.069
Cold swim	$F_{(1,26.1)} = 2.44$	0.130
Rostrocaudal level	$F_{(8,14.6)} =102.03$	0.001***
Inescapable shock * Cold swim	$F_{(1,26.1)} = 0.93$	0.343
Inescapable shock * Rostrocaudal level	$F_{(8,14.6)} = 7.70$	0.001***
Cold swim * Rostrocaudal level	$F_{(8,14.6)} = 2.41$	0.069
Inescapable shock * Cold swim * Rostrocaudal Level	$F_{(8,14.6)} = 2.06$	0.111
**DRI (Unstructured Covariance Structure)**		
Inescapable Shock	$F_{(1,28.0)} = 2.32$	0.139
Cold swim	$F_{(1,28.0)} = 0.39$	0.537
Rostrocaudal level	$F_{(3,26.8)} = 10.86$	0.001***
Inescapable shock * Cold swim	$F_{(1,28.0)} = 0.30$	0.590
Inescapable shock * Rostrocaudal level	$F_{(3,26.8)} = 0.10$	0.961
Cold swim * Rostrocaudal level	$F_{(3,26.8)} = 10.68$	0.001***
Inescapable shock * Cold swim * Rostrocaudal Level	$F_{(3,26.8)} = 0.76$	0.526
**DRC (Unstructured Covariance Structure)**		
Inescapable shock	$F_{(1,28.1)} = 0.01$	0.919
Cold swim	$F_{(1,28.1)} = 0.65$	0.427
Rostrocaudal level	$F_{(3,25.1)} = 1.82$	0.169
Inescapable shock * Cold swim	$F_{(1,28.1)} = 1.38$	0.250
Inescapable shock * Rostrocaudal level	$F_{(3,25.1)} = 1.60$	0.214
Cold swim * Rostrocaudal level	$F_{(3,25.1)} = 3.66$	0.026*
Inescapable shock * Cold swim * Rostrocaudal Level	$F_{(3,25.1)} = 1.22$	0.323

Abbreviations: cDRD, dorsomedial DR (caudal aspect of the dorsal raphe nucleus, dorsal part); cDRV, caudal aspect of the dorsal raphe nucleus, ventral part; DRC, dorsal raphe nucleus, caudal part; DRI, dorsal raphe nucleus, interfascicular part; DRVL, dorsal raphe nucleus, ventrolateral part; rDRD, rostral aspect of the dorsal raphe nucleus, dorsal part; rDRV, rostral aspect of the dorsal raphe nucleus, ventral part; VLPAG, ventrolateral periaqueductal gray.

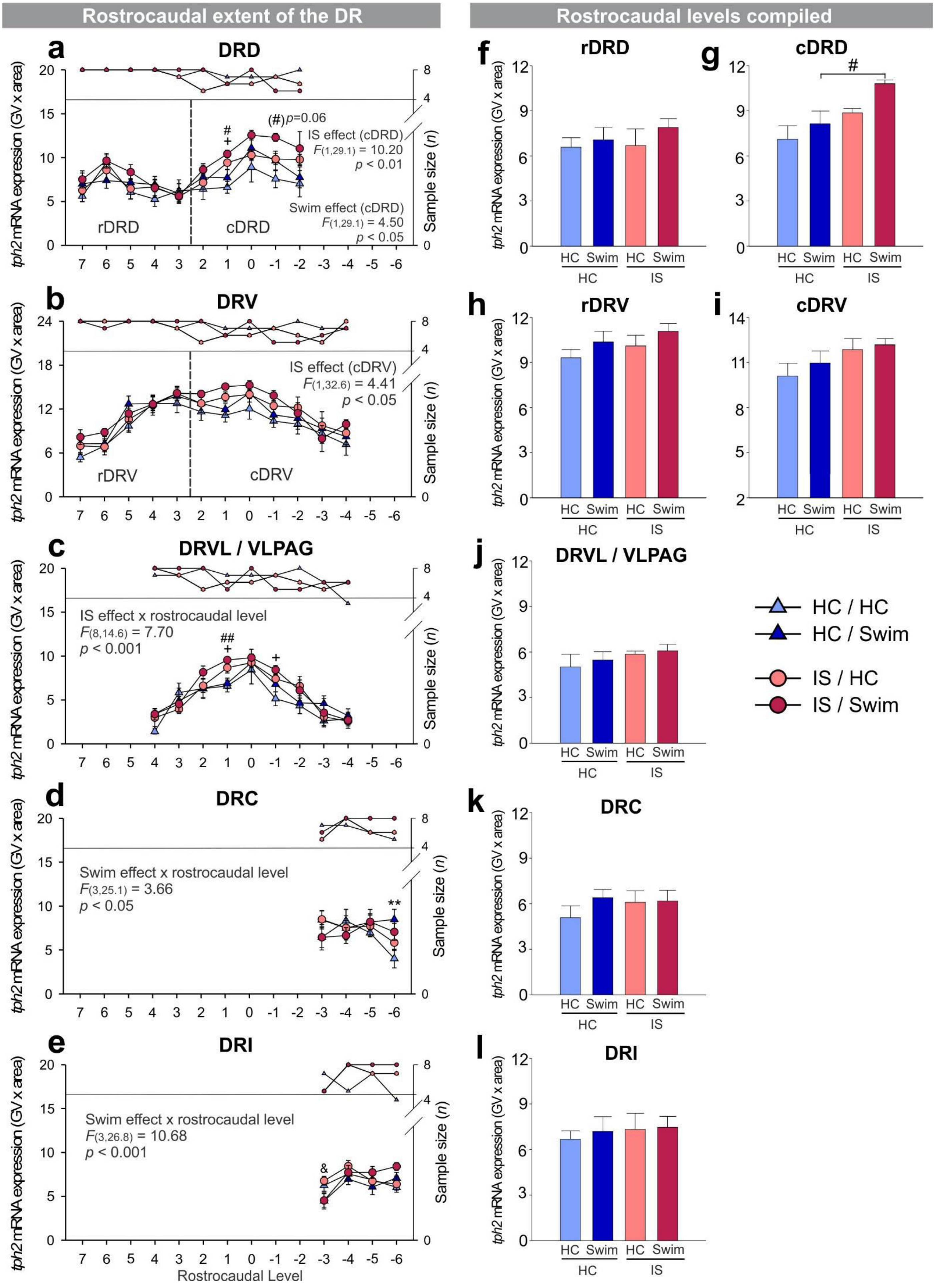
Rostrocaudal extent of the DR
Rostrocaudal levels compiled
a
DRD
(#)p=0.06
IS effect (cDRD)
F(1,29.1) = 10.20
p < 0.01
Swim effect (cDRD)
F(1,29.1) = 4.50
p < 0.05
rDRD
cDRD
b
DRV
IS effect (cDRV)
F(1,32.6) = 4.41
p < 0.05
rDRV
cDRV
c
DRVL / VLPAG
IS effect x rostrocaudal level
F(8,14.6) = 7.70
p < 0.001
d
DRC
Swim effect x rostrocaudal level
F(3,25.1) = 3.66
p < 0.05
e
DRI
Swim effect x rostrocaudal level
F(3,26.8) = 10.68
p < 0.001
Rostrocaudal Level
tph2 mRNA expression (GV x area)
Sample size (n)
f
rDRD
g
cDRD
h
rDRV
i
cDRV
j
DRVL / VLPAG
k
DRC
l
DRI
HC Swim HC Swim
HC IS
HC / HC
HC / Swim
IS / HC
IS / Swim

Supplemental Figure 3. *tph2* mRNA expression following inescapable tail shock (IS) and cold swim stress. Graphs on the left side (**a-e**) represent the mean ± SEM of *tph2* mRNA expression throughout the rostrocaudal extent of each DR subdivision, graphs on the right side (**f-l**) represent the overall *tph2* mRNA expression ± SEM per subdivision (rostrocaudal levels averaged) after home cage (HC) or IS on Day 1, followed by either HC or Swim on Day 2 of *Experiment 3*. All rats were euthanized 4 h after the onset of Swim on Day 2, including unstressed control rats (HC/HC, n = 8), rats that remained in their home cage on Day 1 and were exposed to Swim on Day 2 (HC/Swim, n = 8), rats that received IS on Day 1 and stayed in the home cage on Day 2 (IS/HC, n = 8), and rats that were stressed with IS on Day 1 and with Swim on Day 2 (IS/Swim, n = 8). Graphs illustrate *tph2* mRNA expression in the (**a**) dorsal raphe nucleus, dorsal part (DRD), (**f**) rostral aspect of the DRD (rDRD), (**g**) dorsomedial DR (caudal aspect of the DRD (cDRD)), (**b**) dorsal raphe nucleus, ventral part (DRV), (**h**) rostral aspect of the DRV (rDRV), (**i**) caudal aspect of the DRV (cDRV), (**c**, **j**) dorsal raphe nucleus, ventrolateral part (DRVL) / ventrolateral periaqueductal gray (VLPAG), (**d**, **k**) dorsal raphe nucleus, caudal part (DRC), and (**e**, **l**) dorsal raphe nucleus, interfascicular part (DRI). *Post hoc* comparisons were made using Fisher's least significant difference (LSD) tests; $^{**}p < 0.01$, HC/HC versus HC/Swim; $^{+}p < 0.05$ and $^{++}p < 0.01$, HC/HC versus IS/HC; $^{\#}p < 0.05$, $^{\#\#}p < 0.01$, HC/Swim versus IS/Swim; $^{\&}p < 0.05$, IS/HC versus IS/Swim. *Post hoc* testing was not conducted at a specific rostrocaudal level (7 through -6) when 1 or more groups contained less than half of the full sample size, indicated by the right y-axis. Rostrocaudal levels 7 = -7.580 mm, 6 = -7.664 mm, 5 = -7.748 mm, 4 = -7.832 mm, 3 = -7.916 mm, 2 = -8.000 mm, 1 = -8.084 mm, 0 = -8.168 mm, -1 = -8.252 mm, -2 = -8.336 mm, -3 = -8.420 mm, -4 = -8.504 mm, -5 = -8.588 mm, -6 = -8.672 mm from bregma.

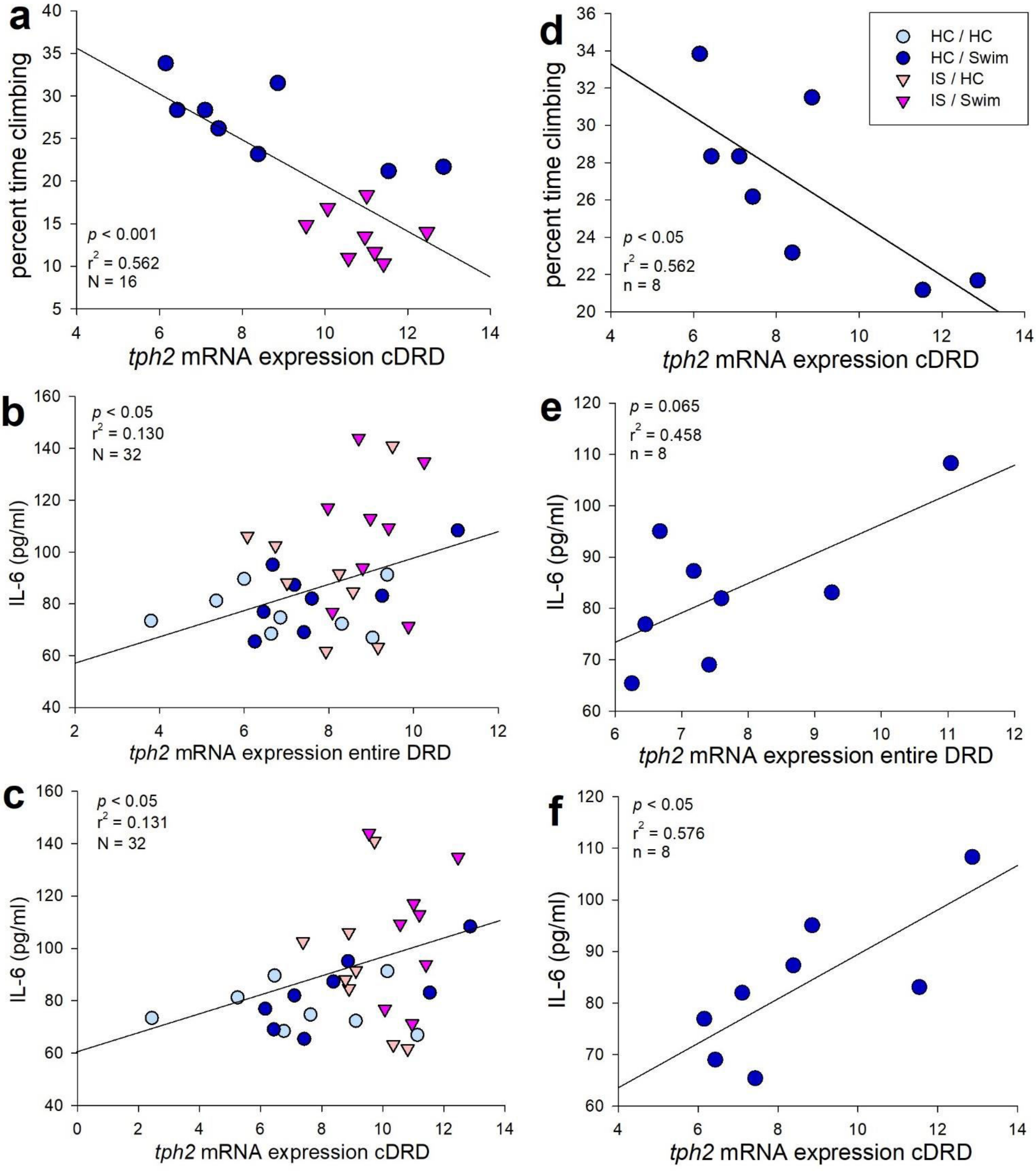

Supplemental Figure 4. Correlations of *tph2* mRNA expression with climbing behavior and plasma interleukin 6 (IL-6) concentrations. (**a**) Inverse correlation between mean *tph2* mRNA expression in the dorsomedial DR (caudal aspect of the dorsal raphe nucleus, dorsal part (cDRD)) of rats that were either exposed to inescapable stress (IS, n = 8) or home cage control conditions (HC, n = 8) on Day 1 and the percent time spent climbing during cold swim on Day 2. (**b**) Positive correlation between mean *tph2* mRNA expression in the entire dorsal raphe nucleus, dorsal part (DRD) and plasma IL-6 concentration. (**c**) Positive correlation between mean *tph2* mRNA in the dorsomedial DR (cDRD) and plasma IL-6. (**d-f**) Graphs illustrating that each of the observed correlations is driven by the HC/Swim group. None of the other treatment groups displayed significant within-group correlations of gene expression and behavior, corticosterone or cytokine levels.

4. Supplemental References

Christianson, J. P., Benison, A. M., Jennings, J., Sandsmark, E. K., Amat, J., Kaufman, R. D., Baratta, M. V., Paul, E. D., Campeau, S., Watkins, L. R., Barth, D. S. and Maier, S. F., (2008). The sensory insular cortex mediates the stress-buffering effects of safety signals but not behavioral control. J Neurosci, 28, 13703-13711.

Christianson, J. P., Jennings, J. H., Ragole, T., Flyer, J. G., Benison, A. M., Barth, D. S., Watkins, L. R. and Maier, S. F., (2011). Safety signals mitigate the consequences of uncontrollable stress via a circuit involving the sensory insular cortex and bed nucleus of the stria terminalis. Biol Psychiatry, 70, 458-464.

Christianson, J. P., Ragole, T., Amat, J., Greenwood, B. N., Strong, P. V., Paul, E. D., Fleshner, M., Watkins, L. R. and Maier, S. F., (2010). 5-hydroxytryptamine 2C receptors in the basolateral amygdala are involved in the expression of anxiety after uncontrollable traumatic stress. Biol Psychiatry, 67, 339-345.

Cryan, J. F., Valentino, R. J. and Lucki, I., (2005). Assessing substrates underlying the behavioral effects of antidepressants using the modified rat forced swimming test. Neuroscience & Biobehavioral Reviews, 29, 547-569.

Dolzani, S. D., Baratta, M. V., Amat, J., Agster, K. L., Saddoris, M. P., Watkins, L. R. and Maier, S. F., (2016). Activation of a Habenulo-Raphe Circuit Is Critical for the Behavioral and Neurochemical Consequences of Uncontrollable Stress in the Male Rat. eNeuro, 3.

Donner, N. C., Montoya, C. D., Lukkes, J. L. and Lowry, C. A., (2012). Chronic non-invasive corticosterone administration abolishes the diurnal pattern of *tph2* expression. Psychoneuroendocrinology, 37, 645-661.

Donner, N. C., Siebler, P. H., Johnson, D. T., Villarreal, M. D., Mani, S., Matti, A. J. and Lowry, C. A., (2016). Serotonergic systems in the balance: CRHR1 and CRHR2 differentially control stress-induced serotonin synthesis. Psychoneuroendocrinology, 63, 178-190.

Drugan, R. C., Hibl, P. T., Kelly, K. J., Dady, K. F., Hale, M. W. and Lowry, C. A., (2013). Prior cold water swim stress alters immobility in the forced swim test and associated activation of serotonergic neurons in the rat dorsal raphe nucleus. Neuroscience, 253, 221-234.

Hale, M. W., Dady, K. F., Evans, A. K. and Lowry, C. A., (2011). Evidence for *in vivo* thermosensitivity of serotonergic neurons in the rat dorsal raphe nucleus and raphe pallidus nucleus implicated in thermoregulatory cooling. Exp Neurol, 227, 264-278.

Hale, M. W., Lukkes, J. L., Dady, K. F., Kelly, K. J., Paul, E. D., Smith, D. G., Raison, C. L. and Lowry, C. A., (2017). Whole-body hyperthermia and a subthreshold dose of citalopram act synergistically to induce antidepressant-like behavioral responses in adolescent rats. Prog Neuropsychopharmacol Biol Psychiatry, 79, 162-168.

Hammack, S. E., Richey, K. J., Watkins, L. R. and Maier, S. F., (2004). Chemical lesion of the bed nucleus of the stria terminalis blocks the behavioral consequences of uncontrollable stress. Behav Neurosci, 118, 443-448.

Kelly, K. J., Donner, N. C., Hale, M. W. and Lowry, C. A., (2011). Swim stress activates serotonergic and nonserotonergic neurons in specific subdivisions of the rat dorsal raphe nucleus in a temperature-dependent manner. Neuroscience, 197, 251-268.

Maier, S. F. and Watkins, L. R., (1998). Stressor controllability, anxiety, and serotonin. Cognitive Therapy Res, 22, 595-613.

Maier, S. F. and Watkins, L. R., (2005). Stressor controllability and learned helplessness: the roles of the dorsal raphe nucleus, serotonin, and corticotropin-releasing factor. Neurosci Biobehav Rev, 29, 829-841.

Malek, Z. S., Dardente, H., Pevet, P. and Raison, S., (2005). Tissue-specific expression of tryptophan hydroxylase mRNAs in the rat midbrain: anatomical evidence and daily profiles. Eur J Neurosci, 22, 895-901.

Malek, Z. S., Pevet, P. and Raison, S., (2004). Circadian change in tryptophan hydroxylase protein levels within the rat intergeniculate leaflets and raphe nuclei. Neuroscience, 125, 749-758.

Malek, Z. S., Sage, D., Pevet, P. and Raison, S., (2007). Daily rhythm of tryptophan hydroxylase-2 messenger ribonucleic acid within raphe neurons is induced by corticoid daily surge and modulated by enhanced locomotor activity. Endocrinology, 148, 5165-5172.

Manjarrez-Gutierrez, G., Martinez-Radilla, K., Boyzo-Montes de Oca, A., Orozco-Suarez, S. and Hernandez-Rodriguez, J., (2012). Increased expression of tryptophan-5-hydroxylase 1, but not 2, in brainstem as a result of intrauterine malnutrition. International Journal of Developmental Neuroscience, 30, 445-450.

Rozeske, R. R., Evans, A. K., Frank, M. G., Watkins, L. R., Lowry, C. A. and Maier, S. F., (2011). Uncontrollable, but not controllable, stress desensitizes 5-HT_{1A} receptors in the dorsal raphe nucleus. J Neurosci, 31, 14107-14115.
